# VEGFD signaling balances stability and activity-dependent structural plasticity of dendrites

**DOI:** 10.1007/s00018-024-05357-2

**Published:** 2024-08-19

**Authors:** Bahar Aksan, Ann-Kristin Kenkel, Jing Yan, Javier Sánchez Romero, Dimitris Missirlis, Daniela Mauceri

**Affiliations:** 1 Department of Neurobiology, Interdisciplinary Centre for Neurosciences (IZN), Heidelberg University, INF 366, 69120 Heidelberg, Germany; 2 Department of Cellular Biophysics, Max-Planck-Institute for Medical Research, Jahnstraße 29, 69120 Heidelberg, Germany; 3 Department Molecular and Cellular Neuroscience, Institute of Anatomy and Cell Biology, University of Marburg, Robert-Koch-Str. 8, 35032 Marburg, Germany

**Keywords:** Dendrite, Neuron, Ezrin, STEP, Structure maintenance, Memory formation, VEGFR3

## Abstract

Mature neurons have stable dendritic architecture, which is essential for the nervous system to operate correctly. The ability to undergo structural plasticity, required to support adaptive processes like memory formation, is still present in mature neurons. It is unclear what molecular and cellular processes control this delicate balance between dendritic structural plasticity and stabilization. Failures in the preservation of optimal dendrite structure due to atrophy or maladaptive plasticity result in abnormal connectivity and are associated with various neurological diseases. Vascular endothelial growth factor D (VEGFD) is critical for the maintenance of mature dendritic trees. Here, we describe how VEGFD affects the neuronal cytoskeleton and demonstrate that VEGFD exerts its effects on dendrite stabilization by influencing the actin cortex and reducing microtubule dynamics. Further, we found that during synaptic activity-induced structural plasticity VEGFD is downregulated. Our findings revealed that VEGFD, acting on its cognate receptor VEGFR3, opposes structural changes by negatively regulating dendrite growth in cultured hippocampal neurons and in vivo in the adult mouse hippocampus with consequences on memory formation. A phosphoproteomic screening identified several regulatory proteins of the cytoskeleton modulated by VEGFD. Among the actin cortex-associated proteins, we found that VEGFD induces dephosphorylation of ezrin at tyrosine 478 via activation of the striatal-enriched protein tyrosine phosphatase (STEP). Activity-triggered structural plasticity of dendrites was impaired by expression of a phospho-deficient mutant ezrin in vitro and in vivo. Thus, VEGFD governs the equilibrium between stabilization and plasticity of dendrites by acting as a molecular brake of structural remodeling.

**Supplementary Information:**

The online version contains supplementary material available at 10.1007/s00018-024-05357-2.

## Introduction

Neurons receive and process synaptic inputs via their dendrites. Therefore, the dendritic architecture of a neuron dictates both its connectivity and the wiring of the network [1, 2]. Dendrites are extremely dynamic during development, but as an organism matures, they gradually stabilize to last for years or even the entirety of its life [3]. Stabilization is essential to preserve the established connections and to ensure proper cognitive functions. A hallmark of many neurological diseases is aberrant connectivity resulting from failure to maintain the dendritic arbor due to pathological remodeling or dendrite atrophy [3–8]. Although mature neurons are largely stable, they retain a certain degree of controlled structural plasticity to enable adaptive processes such as memory formation. Physiological structural changes, typically prompted by synaptic activity, take place both at the level of dendritic spines, hot spots of dynamic events, but also at the level of dendrite arborizations [9, 10].

The primary cytoskeleton components that control the shape of dendrites and spines are actin filaments and microtubules (MTs) [3, 11]. While dendritic spines are primarily enriched in actin, MTs are predominantly localized in the dendritic shaft. In dendrites, MTs are densely packed into networks that grant structural support and the means of transport of proteins and organelles needed distally from the soma [3, 12]. Periodic cortical actin ring structures beneath the dendritic plasma membrane line the inner walls of the membrane and offer mechanical support [11, 13]. Cortical actin ring structures transform into longitudinal bundles of filamentous actin (F-actin) upon synaptic plasticity and play a role in membrane remodeling and protein trafficking [14]. Dendritic filopodia have been observed to nucleate at patches of concentrated F-actin [15, 16].

Both external stimuli, such as synaptic activity or released neurotrophins, as well as intrinsic cell-autonomous programs regulate the structural stability and plasticity of neurons [17, 18]. These inputs influence actin binding proteins (ABPs) and/or microtubule associated proteins (MAPs), which dynamically reshape the cytoskeleton to meet the mechanoelastic requirements of neurons [3].

The specific molecules or factors that promote stabilization and oppose structural plasticity are not fully understood. Vascular endothelial growth factor D (VEGFD), a member of the VEGF family, is a secreted factor known for its role in angiogenesis and lymphangiogenesis [19]. In the nervous system, VEGFD is essential for the preservation of mature dendritic architecture and long-term memory formation [20–22]. In acute neurodegenerative conditions accompanied by loss of dendritic connections, VEGFD supplementation preserved both neuronal integrity and function [23, 24]. The molecular and cellular mechanisms implementing VEGFD-mediated stabilization of dendritic structure and the signaling through which VEGFD imparts instructive signal to the neuronal cytoskeleton are elusive. Moreover, it remains unclear whether VEGFD expression level or downstream signaling may be important in input-driven structural plasticity.

In this study, we characterized the effects of VEGFD on the cytoskeleton in hippocampal neurons and found that VEGFD increases membrane stiffness and negatively affects the growth of microtubules. We demonstrate that VEGFD expression must be reduced for synaptic activity to enable dendritic remodeling, and that VEGFD inhibits activity-induced structural alterations in vitro and in vivo*.* With a combination of morphometric analyses, time-lapse imaging experiments and machine learning-based tracking of dendrites, we describe the effect of VEGFD on dendrite dynamics. Additionally, the actin-plasma membrane linker protein ezrin was found to be a key translator of the VEGFD-mediated stabilization signal to dendrites.

## Materials and methods

### Mice

All animal procedures in this study were carried out in accordance with the ARRIVE guidelines and following approval by the local animal welfare committee (Regierungspräsidium, Karlsruhe, Germany; T-39/22, T-13/21, T-8/18, T-34/16, G-101/21). Male C57Bl/6N mice (Charles River Laboratories) from 8 to 12 weeks of age were group-housed (maximum four per cage) on a 12 h light/dark cycle with food and water ad libitum. Behavioral experiments were done during the light cycle.

### Hippocampal cultures, transfections, and treatments

Hippocampal neurons from P0 C57Bl/6N mice were cultured as described before [22, 25]. DNA transfection was done on 13 days in vitro (DIV) for fluorescence recovery after photobleaching experiments, or DIV 8 for all other experiments by using Lipofectamine 2000 (Invitrogen) as described [20–22]. Experiments were performed after a culturing period of 9–14 DIV. Cells were treated with 100 ng/ml recombinant mouse VEGFD (rVEGFD; R&D Systems), 100 ng/mL recombinant mouse VEGFD (rVEGFD-Alt, Elabscience), 100 ng/mL recombinant mouse VEGFA (rVEGFA; R&D Systems), 100 ng/ml recombinant mouse VEGFC (rVEGFC; Biocat), 50 µM Bicuculline (Tocris Bioscience), 10 µM TC-2153 (Sigma Aldrich) for 1 h, or 1 µM or 10 µM PP2 (Sigma Aldrich) for 1 h or 24 h. Cells were pre-treated for 2 h with 23 nM SAR131675 (Selleckchem) or 2 µg/ml DC101 (Thermo Fisher). Control cells were treated with vehicle only. We routinely assessed viability of the hippocampal neurons in all experiments to exclude possible toxic effects of the treatments. In addition, following overexpression, we monitored the neurons for the possible activation of stress responses, impairment in protein translation and viability.

### Tissue preparation

For immunohistochemistry and X-gal staining, mice were sedated by intraperitoneal injection with 400 mg/kg body weight pentobarbital and transcardially perfused with ice-cold PBS and 4% paraformaldehyde. Brains were collected and post-fixed overnight in 4% paraformaldehyde followed by an incubation in 30% sucrose in PBS for cryoprotection. Brains were embedded in Tissue-Tek (Leica) and cryo-sectioned at a Leica CM1950 cryostat (Leica). For Golgi staining and QRT-PCR, mice were sacrificed with CO_2_ and decapitated. Whole brains (for Golgi staining) or CA1 regions of the hippocampus (for QRT-PCR) were isolated and shock-frozen in liquid nitrogen.

### Plasmids

pGW1-CAG > EB3-GFP, pRRL-CMV > lifeact-GFP, pAAV-CMV > VEGFD-HA, pAAV-CMV > LacZ-Flag, pAAV-CMV > hrGFP and pAAV-CaMKII > jRGECO1alpha-NLS have been characterized before [20, 26–30]. The cDNA encoding mouse VEGFD, LacZ and mouse STEP61 were each subcloned into a pAAV vector, downstream of the activity-dependent ESARE promoter [31, 32] and C-terminally tagged with HA. The same vector also includes a human synapsin (hsyn) promoter driving the expression of tDimer.

The cDNA sequences of human WT and a phospho-deficient mutant ezrin, provided by Peter A. Greer and Bruce E. Elliott [33], were subcloned into a pAAV vector under the control of hsyn promoter followed by a C-terminal Flag-tag. A control vector encoding LacZ C-terminally tagged with HA under the control of hsyn promoter served as control.

### Recombinant adeno-associated viruses (rAAVs)

Viral particles were produced and purified as described previously [34–36]. Infections of primary mouse hippocampal neurons with rAAVs were done at DIV 3. Infection efficiencies were determined immunocytochemically and by immunoblotting using antibodies to the appropriate tag.

### Fluorescence recovery after photobleaching and analysis

Fluorescence recovery after photobleaching (FRAP) was performed as described before [26, 30]. Briefly, primary hippocampal neurons were transfected at DIV 13 with lifeact-GFP. FRAP was performed 5, 15 or 30 min after treatment with 100 ng/ml rVEGFD at DIV 14, a timepoint in which spines are mostly found in a mature mushroom type shape. Image acquisition and bleaching were performed using a Leica Sp8 confocal laser scanning microscope with a 63 × water immersion objective and 6 × optical zoom at a resolution of 1024 × 1024 pixels with 0.029 µm/pixel in a HEPES-buffered saline solution (HBS) containing: 140 mM NaCl, 5.3 mM KCl, 1.0 mM MgCl_2_, 2.0 mM CaCl_2_, 10 mM HEPES, 1.0 mM glycine, 35.6 mM D-glucose, and 0.5 mM Na-pyruvate. Time-lapse images were acquired at an interval of 1.48 s. Baseline was recorded for three scans before bleaching with five scans (total bleach time: 7.4 s) at a laser intensity of 70%. Immediately after bleaching, recording was continued for another 60 frames (total time: 100.64 s). Per neuron, FRAP was performed on three spines located on different dendrites far from each other to exclude off-target bleach effects. FRAP images were analyzed using ImageJ. After background subtraction (rolling ball radius: 50 pixels), fluorescence intensities in the bleached spine and in a small area of an unrelated dendrite in the same frame were measured using the oval tool. Fluorescence from spines were normalized to the intensities of dendrites of the same frame and on the average prebleach intensity. Half-time of recovery, stable and mobile fraction were calculated by fitting the normalized intensities to the following equation using Graphpad Prism 8 (GraphPad Software, Inc.) as described before [26]:

$${\text{Y}} = {\text{Y}}0 + \left( {{\text{Plateau}} - {\text{Y}}0} \right) \times \left( {1 - {\text{e}}\left( { - {\text{K}} \times {\text{x}}} \right)} \right),$$

X = time; Y0 = first intensity value of postbleach; Y = percentage of recovery, starts with Y0; K = constant rate.

### Atomic force microscopy

Young’s Modulus (kPa) was determined using atomic force microscopy (AFM). Force spectroscopy measurements were performed on a Nanowizard 3 AFM (JPK Instruments) mounted on an inverted optical microscope (Zeiss), equipped with a 40 × objective (Zeiss) in a temperature- and CO_2_- controlled chamber. Colloidal (spherical) probes with 2 μm diameter made of SiO_2_ were used (sQube). The spring constant of the cantilever ranged between 0.2 and 1.0 N/m and was determined prior to each experiment using the thermal noise method. Measurements were performed in cell culture medium at DIV 10 on the soma and proximal dendrites. The indentation depth was kept below 1 µm, to avoid contributions from the underlying substrate. Typically, 30 curves/cell and 30 cells were measured per independent experiment. Force-distance curves were analyzed using the Hertz model for a spherical tip indenting a flat sample. The force in this case is given by: $F = \frac{{4{\text{ E}}}}{{3{ }\left( {1 - v^{2} } \right)}}\sqrt R \delta^{\frac{2}{3}}$

E = Young’s modulus; v = Poisson ratio, R = radius of the sphere; δ = indentation depth. The Poisson ratio was taken as 0.5, as typically done for cells. The analysis was performed by a custom-made script using Python and the force-distance curve was fit using a least-squares method.

### Time-lapse of EB3-comets and analyses

Time-lapse images of EB3-GFP movements, appearing as fluorescent comets, in dendrites of transfected cells at DIV 10 were acquired every 2.57 s using a Leica Sp8 confocal laser scanning microscope with a 63 × water immersion objective and 4 × optical zoom at a resolution of 1024 × 1024 pixels with 0.043 µm/pixel in a HEPES-buffered saline solution (HBS) containing: 140 mM NaCl, 2.5 mM KCl, 1.0 mM MgCl_2_, 2.0 mM CaCl_2_, 10 mM HEPES, 1.0 mM glycine, 35.6 mM D-glucose, and 0.5 mM Na-pyruvate. Only secondary dendrites within similar distance from the soma were analyzed. After 5 min of baseline recording, neuronal cultures were treated with the indicated drugs and imaging continued for 5 min. For analysis, images were subjected to standard average subtraction and convolution to reduce noise as described before [37]. Individual dendrites were traced manually, and kymographs were generated using the KymoResliceWide ImageJ plugin [37]. EB3 comets were manually traced. Only motile (non-vertical) comets visible for at least 10 s were considered. EB3 comet lifetime refers to the time an EB3 comet spent traveling and was assessed by measuring the length of time that an EB3 comet was visible on a kymograph. The speed of EB3 comets was calculated by dividing the distances travelled by time spent traveling (comet lifetime). Number of EB3 comets was counted per 100 µm and min. All imaging processing was performed in ImageJ. Data processing was performed in R 3.6.3 (R Foundation for Statistical Computing).

### Phospho-antibody array

Cultured neurons treated for 15 min or 2 h with 100 ng/ml rVEGFD were harvested and subjected to an ELISA-based cytoskeleton phospho-antibody array containing 143 different antibodies in parallel to untreated control cultured neurons (FullMoon Biosystems) according to manufacturer’s instructions. For data analysis, background was subtracted by the values obtained for each antibody and each sample was additionally normalized for the value obtained for beta-actin for protein content.

### Real-time quantitative polymerase chain reaction (QRT-PCR)

Total RNA was extracted from primary mouse hippocampal neurons or mouse CA1 using the RNeasy Mini Kit (QIAGEN) including an optional DNase I treatment at room temperature for 15 min according to the manufacturer’s instructions (QIAGEN).

RNA from cultured neurons or from CA1 was reverse transcribed into first-strand cDNA using a high-capacity cDNA reverse transcription kit (Applied Biosystems). QRT-PCR was performed on a StepOne Plus real-time PCR system using the following TaqMan gene expression assays (Applied Biosystems):

*vegfd* (Mm00438963_m1), *cfos* (Mm00487425_m1), flt4*/VEGFR3* (Mm01292618_m1), *kdr/VEGFR2* (Mm00440099_m1), and *gusb* (Mm00446953_m1) as an endogenous control gene for normalization.

### Western blotting

Primary hippocampal cultures were harvested directly in Laemmli buffer (30% glycerol, 4% SDS, 160 mM Tris–HCl pH 6.8, 0.02% bromophenol blue, 10 mM DTT) and boiled for 10 min at 95 °C. Standard protocols for western blot were used.

The following primary antibodies were used: goat anti-VEGFD (1:600 in 5% BSA; AF469, R&D Systems), mouse anti-ezrin (1:1000 in 5% BSA; sc-58758, Santa Cruz Biotechnology), mouse anti-phospho-(Y478) ezrin (1:1500 in 5% BSA; TA326188, OriGene Technologies), mouse anti-STEP (1:1000 in 5% milk; 4396, Cell Signaling Technology), rabbit anti-non phospho-(S221) STEP (1:3000 in 5% BSA; 5659, Cell Signaling Technology), rabbit anti-phospho-(Y1472) GluN2B (1:1000 in 5% BSA; 4208, NEB), mouse anti-phospho-(T202/Y204) p44/ 42 ERK (1:2000 in 5% BSA; 9106, NEB), mouse anti-phospho-(T180/Y182) p38 (1:750 in 5% milk; 612,288, BD Biosciences), rabbit anti-phospho-(S473) Akt (1:1000 in 5% BSA; 9271, Cell Signaling), rabbit anti-phospho-(S172) NFATc1 (1:1000 in 5% milk; PA5-64,696, Invitrogen), rabbit anti-phospho-(Y416) Src family (1:1000 in 5% BSA; 6943, Cell Signaling), goat anti-VEGFR3 (1:1000 in 5% milk; AF743, R&D Systems), rabbit anti-VEGFR2 (1:500 in 5% milk; 2479, R&D Systems), mouse anti-Flag (1:5000 in 5% milk; F3165, Sigma Aldrich), mouse anti-dsRed (1:1000 in 5% BSA; 632,496, Clontech Laboratories), mouse anti-HA (1:7500 in 5% milk; MMS-101R, Covance), rabbit anti-cFos (1:2000 in 5% BSA; sc-52, Santa Cruz Biotechnology), rabbit anti-gapdh (1:20,000 in 5% BSA; 2118, Cell Signaling Technology), mouse anti-tubulin (1:400,000 in 5% milk; T9026, Merck Millipore). The following antibodies were used 1:5000 in 5% milk as secondary antibodies: goat anti-mouse IgG (H + L) peroxidase (115-035-003, AffiniPure Jackson Immuno Research), goat anti-rabbit IgG (H + L) peroxidase (111-035-144, AffiniPure Jackson Immuno Research) and donkey anti-goat IgG (H + L) peroxidase (705-035-147, AffiniPure Jackson Immuno Research). Membranes were developed with enhanced chemiluminescence (ECL) solution (BioRad) and the ChemiDoc imaging system (Bio-Rad). Relative protein content was normalized to tubulin and control sample. Images were generated using a Chemidoc Imaging system (Biorad).

For fluorescent western blotting, the Odyssey Infrared Imaging System (LI-COR Biosciences) was used according to the manufacturer’s protocol using the following secondary antibodies: IRDye 680LT donkey anti-mouse and IRDye 800CW donkey anti-rabbit (1:15,000 in Odyssey blocking buffer, LI-COR Biosciences). Images of the blots were acquired with the Image Studio Software 4.0 (LI-COR Biosciences).

### Immunocytochemistry

Cultured neurons were fixed with a 4% paraformaldehyde and 4% sucrose solution for 20 min. For immunostaining, antibodies were applied in GDB buffer, containing 16.5 mM phosphate buffer [pH7.4], 0.1% gelatine, 0.3% Triton X-100, and 0.45 M NaCl. The following primary antibodies were applied overnight at 4 °C: mouse anti-HA (1:1000; MMS-101R, Covance), mouse anti-Flag (1:200; F3165, Sigma Aldrich), and rabbit anti-MAP2 (1:1000; 188,002, Synaptic Systems). Secondary antibody incubation was done for 1 h at room temperature. The following secondary antibodies were used: goat anti-mouse IgG (H + L) Alexa Fluor 488 (1:1000; A11001, Life Technologies), goat anti-mouse IgG (H + L) Alexa Fluor 594 (1:1000; A11005, Life Technologies), and goat-anti-rabbit IgG (H + L) Alexa Fluor 488 (1:1000; A11008, Life Technologies). Hoechst was used to label nuclei (1:6000 in PBS; Serva Electrophoresis) for 5 min.

Proximity ligation assay was performed using a Duolink® In Situ Red Starter Kit Mouse/Rabbit and Mouse/Goat (Sigma Aldrich) according to the manufacturer’s protocol. Briefly, total protein and phosphorylated form of the protein are targeted by an antibody pair from different species. Secondary antibodies coupled to complementary oligonucleotides bind to the primary antibody pair and come into proximity on phospho-proteins so that complementary strands can be hybridized by a ligase followed by a PCR amplification with fluorescent probes. The following primary antibodies were used: mouse anti-ezrin (1:50; sc-58758, Santa Cruz), rabbit anti-phospho-(Y478) ezrin (1:50; TA326188, OriGene Technologies), goat anti-VEGFR3 (1:100; AF743, R&D Systems), and mouse anti-phospho-tyrosine (1:100; 309,301, BioLegend).

Images were acquired with a Leica DM IRBE inverted fluorescent microscope with a 40 × oil immersion objective and a Spot Insight FireWire 2 camera with VisiView software (Visitron Systems) or with a Leica Sp8 confocal laser scanning microscope and a 63 × oil immersion objective. PLA clusters were counted in ImageJ and normalized to Hoechst-labeled cell number.

### Immunohistochemistry

Free-floating coronal brain sections of 30 µm thickness from perfused mice were blocked in a solution of 8% normal goat serum and 0.3% Triton X-100 in PBS for one hour at room temperature. Antibodies were diluted in a solution of 2% normal goat serum and 0.3% Triton X-100 in PBS. The following primary antibodies were used overnight at 4 °C: mouse anti-HA antibody (1:1000; MMS-101R, Covance) and mouse anti-Flag (1:100; F3165, Sigma Aldrich). Goat anti-mouse IgG (H + L) Alexa Fluor 488 (1:1000; A11001, Life Technologies) was used as secondary antibody for 90 min at room temperature. Hoechst was used to label nuclei (1:6000 in PBS; Serva Electrophoresis) for 5 min. Images were acquired at a Leica Sp8 confocal laser scanning microscope with a 5 × objective.

### X-gal staining

For X-gal staining, free-floating coronal brain sections of 30 µm thickness from perfused mice were washed with a detergent rinse (44.27 mM KH_2_PO_4_, 55.73 mM Na_2_HPO_4_, 2 mM MgCl_2_, 0.01% (w/v) sodium deoxycholate, 0.02% (v/v) IGEPAL CA-630) for 10 min at room temperature, before staining with an X-gal solution (5 mM K_3_[Fe(CN)_6_], 5 mM K_4_[Fe(CN)_6_], 1 mg/ml X-gal (pre-dissolved in DMSO; B4252, Sigma Aldrich) in detergent rinse) for 30 min at room temperature. Images were acquired with a Nikon Eclipse Ni-E widefield microscope with a 5 × objective.

### ELISA VEGFD

Mouse VEGFD levels in homogenates and medium of cultured neurons were measured using a specific ELISA kit (Cusabio) according to the manufacturer’s protocol. In brief, following treatments, medium was collected, and, after centrifugation at 1000 g for 20 min, supernatants aliquoted and snap frozen. Neurons were harvested and lysed using a glass dounce homogenizer in a buffer containing 0.32 M sucrose, 1 mM HEPES, 1 mM MgCl_2_, 0.1 mM PMSF, 1 mM NaHCO_3_, 1 mM NaF, cOmplete™ Mini EDTA-free protease-inhibitor-cocktail (Roche), phosphatase inhibitor cocktail 2 (Sigma Aldrich). Supernatants were collected after centrifugation at 1000 g for 15 min. Samples were analyzed in duplicate, and a calibration standard curve was prepared for each assay. We next generated a four parameter logistic (4-PL) curve fit with Prism software and calculated VEGFD concentrations.

### Morphometric analysis of fixed dendrites

Dendrites in cultured neurons were visualized via hrGFP-transfection and images acquired at DIV 10 or DIV 14 using a Leica Sp8 confocal laser scanning microscope with a 63 × oil immersion objective. All images were obtained as a z series projection with 0.5 µm intervals and at a resolution of 1024 × 1024 pixels with 0.172 µm/pixel.

Total dendritic length and Sholl analysis [38] were performed using the Simple Neurite Tracer ImageJ plugin as described [10, 22, 23]. Sholl interval was set to 5 µm. Total number of intersections was defined by the sum of all crossings between the traced dendrites and the Sholl rings used for Sholl analysis up to a radius of 150 µm.

### Golgi staining and analyses

Golgi staining was performed on 100-μm-thick coronal slices of the mouse brain using a Rapid Golgi Stain Kit (FD Neuro Technologies) according to the manufacturer’s protocol and as described before [20, 23]. Golgi-stained CA1 neurons were acquired with a 20 × objective mounted on a Nikon Eclipse 90i upright automated microscope as z series projections in 5 μm intervals. Dendrites were manually traced using the Simple Neurite Tracer ImageJ plugin.

### Neurite length analysis over time

Neurons were transfected at DIV 8 with hrGFP to visualize dendrites and treated at DIV 9 after robust onset of GFP expression. Afterward, neurons were kept in an Incucyte® S3 Live-Cell Analysis System (Sartorius) and images were acquired every 4 h for a total of 24 h in the green channel (excitation: 441—481 nm, emission: 502—544 nm, acquisition time: 300 ms) with a 20 × objective. 16 images (875 × 645 µm/image) per well of a size of 1.9 cm^2^, containing ca. 0.18 × 10^6^ cells, were acquired. Total neurite length of each well was measured with the Incucyte® Neurotrack Analysis Software Module (Sartorius) using the following parameters: Cell-Body Cluster Segmentation: Top-Hat, radius: 20 µm, threshold: 1.5 GCU, min cell width: 11 µm, neurite course sensitivity: 10, neurite fine sensitivity: 0.5. The total neurite length was always normalized per well to the first timepoint after treatment start.

### Live imaging and machine-learning based segmentation of dynamic dendrites

Cultured neurons were transfected at DIV 8 with hrGFP to visualize dendrites and treated at DIV 9 with 100 ng/ml rVEGFD, 50 µM Bic or 100 ng/ml rVEGFD and 50 µM Bic in combination. After setting up of the system, neurons were imaged every hour for a total of 12 h at an inverted Nikon Eclipse Ti2 widefield microscope made for high content screening in the green channel (emission wavelength: 520 nm) with a 20 × objective and 1.5 × optical zoom equipped with a Nikon DS-Qi2 camera. All images were obtained as a z series projection with 1 µm intervals and at a resolution of 2424 × 2424 pixels with 0.24 µm/pixel. Perfect focus system was used to reduce focus drift. Neurons were kept in a CO_2_ and temperature controlled dark chamber during imaging.

A machine-learning based script developed by KARMENscience® (karmenscience.ai; Croatia, EU) and trained extensively by a scientist with experience in dendrite tracing, was used to automatically segment dendrites in time-lapse videos. Only dendrites, and not axons, belonging to the same neuron were traced.

In the first step of the pipeline, z-stacks were superposed to one image per timepoint. For each neuron at its first timepoint, soma and dendrites were segmented. For each subsequent image, the previously marked dendrites were inherited, and changes in comparison to the previous image were recognized automatically. Each segmentation was manually checked for errors and if required, adjusted manually.

KARMENscience® developed an algorithm that recognizes which dendrite is linked to which dendrite across all timepoints and delivers a table with length measurements for each dendrite at every timepoint, as well as calculations of the number of all, new or eliminated dendrites.

### Stereotaxic delivery of rAAVs

Stereotaxic injections of rAAVs into the dorsal hippocampus were performed as described before [39]. Briefly, rAAVs were injected at the following coordinates relative to the bregma: − 2 mm anteroposterior; ± 1.5 mm mediolateral; −1.7, −1.9, and −2.1 mm dorsoventral. A total volume of 500 nl virus in PBS was injected at 200 nl/min per spot. Following injections, the needle was left in place for 60 s.

### Contextual and cued fear conditioning

Contextual and cued fear conditioning was conducted as described before [10]. Briefly, mice were placed in a conditioning chamber and received four 1 s foot shocks (0.6 mA) followed by a 30 s tone (5000 Hz, 85 dB). Before the first shock and between foot shocks, mice were allowed to freely explore the chamber for 1 min each. Control mice were also placed in the chamber but did not receive the shocks or tones. Freezing behavior was recorded and analyzed. For auditory-cued fear retention, mice were placed in a novel chamber and two tones lasting for 30 s were delivered. Freezing behavior was recorded and analyzed. Mice were sacrificed two days later. All behavioral experiments were performed by the same experimenter in awake mice during the light phase. Mice were acclimatized to the experimenter and the room prior to behavioral experiments.

### Statistical analysis

All graphs represent mean ± SEM. Statistical analyses were performed using GraphPad Prism 9 (GraphPad Software, Inc.). All data were tested for normality distribution using the Shapiro–Wilk normality test (alpha = 0.05). For the comparison of an experimental condition to a normalized control, one sample t-test (parametric) or Wilcoxon signed-rank test (nonparametric) were performed. Two-tailed unpaired Student’s t-test (parametric), two-tailed unpaired Mann–Whitney test (nonparametric), or multiple t-tests were used to compare two groups. More than two conditions were analyzed with One-way ANOVA (parametric) or Kruskal–Wallis test (nonparametric) followed by an appropriate post hoc test. For comparisons of multiple conditions with two independent variables, Two-way ANOVA followed by an appropriate post hoc test was performed. Details on the statistical tests and sample sizes are indicated in the respective figure legends.

## Results

### VEGFD modulates cortical actin and microtubules

VEGFD is a known modulator of cellular morphology, particularly of neural cells, but its effects on cytoskeleton elements are unclear [20–24, 40–43].

**Fig. 1:**
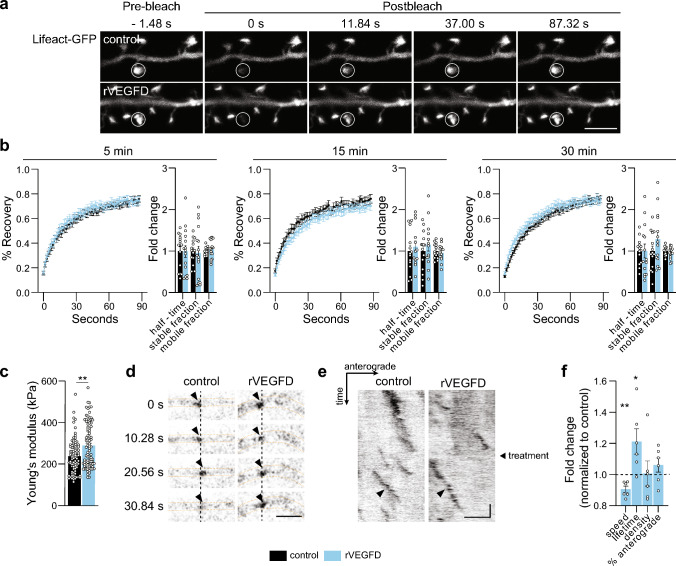
Characterization of the effects of VEGFD on neuronal cytoskeleton. **a**, **b** Fluorescence recovery after photobleaching (FRAP) analysis of actin turnover in spines of Lifeact-GFP-transfected cultured hippocampal neurons with or without 5, 15 or 30 min rVEGFD treatment. **a** Representative images of FRAP time-lapse series of neurons with or without 15 min rVEGFD treatment. Bleached spine is marked with a circle. Frames before (−1.48 s) and after bleaching (0 s–87.32 s) are shown. Scale bar = 5 µm. **b** Lifeact-GFP FRAP recovery curves from multiple spines for each condition normalized to average pre-bleach fluorescence and quantifications of half-time, stable and mobile fraction normalized to respective untreated control. The half-time is the duration required for half of the intensity to recover. The stable and mobile fraction refer to the non-recovered and recovered fractions, comprising slow and fast exchanging actin molecules, respectively. Two-tailed unpaired Student’s t-test or Mann–Whitney test. N = 13–16 neurons from 4 independent culture preparations, 1–3 spines per neuron, 31–37 spines/condition in total. **c** Atomic force microscopy measurements on the soma and proximal dendrites of cultured hippocampal neurons with or without rVEGFD treatment. Mann–Whitney test. N = 88–90 neurons from 3 independent culture preparations. **d**–**f** Analysis of microtubule (MT) dynamics in microtubule plus-end-binding protein 3 (EB3)-GFP-transfected cultured hippocampal neurons. After 5 min of EB3-GFP comet baseline recording, neurons were treated with rVEGFD or vehicle and imaging continued for 5 min. **d** Representative timeseries of EB3-GFP comets (marked with arrowheads) moving along a dendrite in neurons treated with rVEGFD or vehicle. Vertical dashed line helps to distinguish the trajectory of a comet. Scale bar = 2 µm. **e** Representative kymographs of EB3-GFP motility from images shown in (**d**). Arrowheads mark EB3-GFP comets that are indicated in (**d**). Start of treatment is marked on the right. Scale bar = 2 µm, 60 s. **f** EB3-GFP comet speed, lifetime, density, and percentage of anterograde comet movements normalized on baseline and respective untreated control. One sample t-test. N = 6 independent culture preparations. 10 neurons/condition in total, on average 5–8 dendrites per neuron. Graphs represent mean ± SEM. Dots represent single values. **p < 0.01; *p < 0.05

Thus, to investigate the impact of VEGFD on the neuronal cytoskeleton, we first characterized its potential effect on actin localized in dendritic spines in cultured hippocampal neurons. We used fluorescence recovery after photobleaching (FRAP) of lifeact-green fluorescent protein (GFP) to monitor actin dynamics [26, 30]. When expressed in cultured neurons, lifeact-GFP stains actin and accumulates in dendritic spines, reflecting the distribution of endogenous actin [26, 30]. Consistent with previous indications that VEGFD regulates dendrites but not dendritic spines shape or density [20, 42], we found no difference in the fluorescence recovery of lifeact-GFP after photobleaching in spines between control neurons and neurons exposed to recombinant VEGFD (rVEGFD) at any of the time points analyzed, indicative of no change in actin assembly in spines (Fig. 1a, b).

Next, we used atomic force microscopy (AFM) to measure the elasticity of the plasma membrane [44]- determined by the cortical actin and its attachment to the membrane—of neurons treated with rVEGFD [45, 46]. In agreement with previous observations carried out on cancer cells [47], AFM revealed that VEGFD increases the stiffness of cultured neurons (Fig. 1c).

Finally, as VEGFD regulates dendritic architecture [20–23, 40–42], we assessed the influence of VEGFD on microtubules (MTs) which are the prevalent cytoskeleton element granting stability to dendritic shafts [48–50]. We expressed microtubule plus-end-binding (EB) protein EB3 conjugated to GFP in cultured neurons. EB3-GFP binds to the ends of growing MTs and appears as comet-like structures in time-lapse microscopy, allowing the visualization and analyses of MTs dynamics [28, 49] (Fig. 1d, e). In rVEGFD-treated neurons, EB3-labeled comets travelled slower and had an increased lifetime (Fig. 1f). Comet density and direction of MT growth was not affected (Fig. 1f).

Taken together, our data suggest that VEGFD exerts its structural stabilization function by influencing cortical actin and MTs.

### Reduction of VEGFD expression is necessary to allow synaptic activity-dependent dendritic remodeling in cultured neurons

**Fig. 2:**
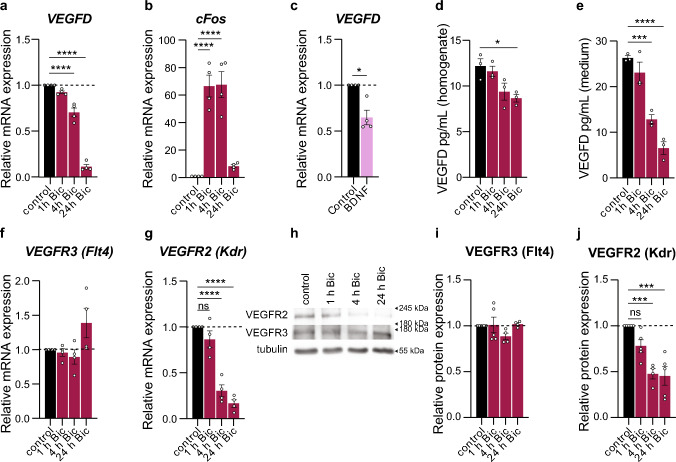
VEGFD expression is reduced following long-lasting increased synaptic activity. **a**–**b** QRT-PCR analysis of *VEGFD* (**a**) or *cFos* (**b**) mRNA expression in cultured hippocampal neurons with or without treatment of bicuculline (Bic) for 1, 4 or 24 h. One-way ANOVA followed by Dunnett’s post hoc test. N = 4 independent culture preparations. **c** QRT-PCR analysis of *VEGFD* mRNA expression in cultured hippocampal neurons with or without BDNF treatment for 4 h. One sample t-test. N = 4 independent culture preparations. **d**, **e** ELISA quantification of VEGFD protein in lysates (**d**) or medium (**e**) of cultured hippocampal neurons with or without treatment of Bic for 1, 4 or 24 h. One-way ANOVA followed by Dunnett’s post hoc test. N = 3 independent culture preparations. **f**, **g** QRT-PCR analysis of *VEGFR3 (Flt4)* (**f**) or *VEGFR2 (Kdr)* (**g**) mRNA expression in cultured hippocampal neurons with or without treatment of Bic for 1, 4 or 24 h. One-way ANOVA followed by Dunnett’s post hoc test. N = 4 independent culture preparations. **h**–**j** Immunoblot analyses of VEGFR3 (Flt4) and VEGFR2 (Kdr) protein expression in cultured hippocampal neurons with or without treatment of Bic for 1, 4 or 24 h. **h** Representative immunoblots of VEGFR2, VEGFR3, and tubulin. VEGFR3 (**i**) or VEGFR2 (**j**) levels normalized to tubulin expression and control. One-way ANOVA followed by Dunnett’s post hoc test. N = 4–5 independent culture preparations. Graphs represent mean ± SEM. Dots represent single values. ****p < 0.0001; ***p < 0.001; *p < 0.05; ns p > 0.05

Synaptic activity is a key player in the regulation of neuronal structure [5, 7, 51]. The gamma-aminobutyric acid-A-receptor antagonist bicuculline (Bic) relieves the inhibitory tone of the cultured neurons and triggers bursts of action potentials [52]. Surprisingly, we detected a time-dependent reduction of *VEGFD* mRNA levels as a result of the Bic-triggered increased synaptic activity (Fig. 2a). As expected, mRNA levels of *cFos*, a control marker for neuronal activity [53], were increased following Bic treatment (Fig. 2b). To corroborate our findings, we employed a different strategy independently of Bic to boost synaptic activity and monitor VEGFD expression. Brain derived neurotrophic factor (BDNF) administration increases synaptic activity [54] and neuronal remodeling [55, 56] of cultured hippocampal neurons. In agreement with our observations obtained using Bic, BDNF exposure also prompted a reduction of *VEGFD* mRNA levels (Fig. 2c). Next, we measured VEGFD protein level via ELISA and we measured a significant reduction of VEGFD protein in both lysates and medium of Bic-treated hippocampal cultures (Fig. 2d, e). VEGFD has two possible receptors, VEGFR2 (also named Kdr) and VEGFR3 (also known as Flt4). We monitored the expression of both receptors after Bic stimulation. While VEGFR3 mRNA and protein levels remained constant after Bic treatment, VEGFR2 significantly decreased (Fig. 2f–j).

As VEGFD levels are key in upholding neuronal structural integrity and protecting dendrites from damage [20, 22–24], we hypothesized that the detected activity-triggered downregulation of VEGFD may be necessary to structural remodeling.

**Fig. 3:**
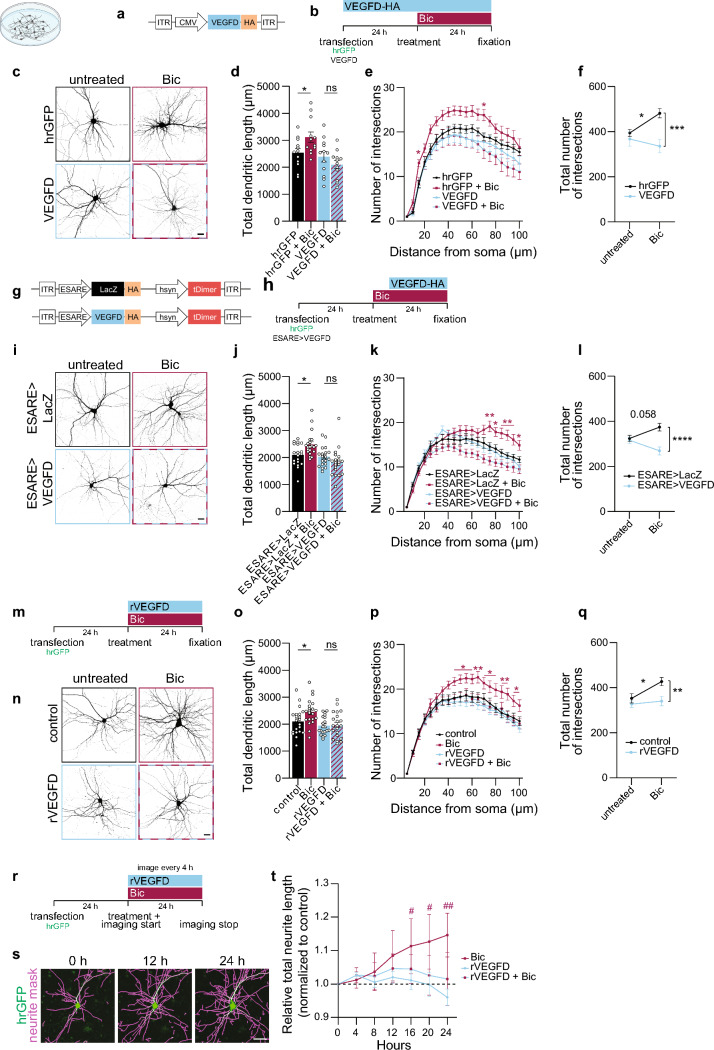
Reduced VEGFD expression is necessary to allow dendritic remodeling in cultured neurons. **a**–**f** Morphometric analyses of cultured hippocampal neurons transfected with hrGFP (vector) and/or VEGFD-HA, with or without bicuculline (Bic) treatment for 24 h. **a** Schema of the construct used. **b** Schema of the experimental setup. **c** Representative images of neurons transfected and treated as indicated. hrGFP was used to visualize neurons. Scale bar = 20 µm. **d**–**f** Total dendritic length (**d**), Sholl analysis (**e**) and total number of intersections (VEGFD vs VEGFD + Bic, p = 0.7299) (**f**) of neurons transfected and treated as indicated. One and Two-way ANOVA followed by Bonferroni’s post hoc test (**d**, **f**), or Dunnett’s post hoc test (**e**). N = 12 neurons from 3 independent culture preparations. **g**–**l** Morphometric analyses of cultured hippocampal neurons co-transfected with hrGFP and ESARE > VEGFD-HA or ESARE > LacZ-HA, with or without Bic treatment for 24 h. **g** Schema of the constructs used. **h** Schema of the experimental setup. **i** Representative images of neurons transfected and treated as indicated. hrGFP was used to visualize neurons. Scale bar = 20 µm. **j**–**l** Total dendritic length (**j**), Sholl analysis (**k**) and total number of intersections (ESARE > VEGFD vs ESARE > VEGFD + Bic, p = 0.0826) (**l**) of neurons transfected and treated as indicated. Kruskal–Wallis test followed by Dunn’s (**j**), or Two-way ANOVA followed by Dunnett’s post hoc test (**k**) or Bonferroni’s post hoc test (**l**). N = 19–21 neurons from 4 independent culture preparations. **m**–**q** Morphometric analyses of cultured hippocampal neurons transfected with hrGFP, with or without rVEGFD or Bic treatment, or both for 24 h. **m** Schema of the experimental setup. **n** Representative images of neurons treated as indicated. hrGFP was used to visualize neurons. Scale bar = 20 µm. **o**–**q** Total dendritic length (**o**), Sholl analysis (**p**) and total number of intersections (rVEGFD vs rVEGFD + Bic, p > 0.9999) (**q**) of neurons treated as indicated. One- and Two-way ANOVA followed by Bonferroni’s post hoc test (**o**, **q**), or Dunnett’s post hoc test (**p**). N = 20–21 neurons from 4 independent culture preparations. **r**–**t** Time-lapse imaging of neurite length for 24 h of hrGFP-transfected cultured hippocampal neurons, with or without rVEGFD or Bic treatment, or both. **r** Schema of the experimental setup. **s** Representative images of a hrGFP-expressing neuron at indicated timepoints overlaid with an automatically generated neurite mask used to measure neurite length. Scale bar = 50 µm. **t** Relative total neurite length over time normalized on the first timepoint and untreated control. Two-way ANOVA with repeated measures followed by Dunnett’s post hoc test for comparisons to basal and control values. N = 3 independent cultures. Graphs represent mean ± SEM. Dots represent single values. Asterisks (*) refer to statistical comparisons between conditions and hashtags (#) to comparisons to basal values per condition. ****p < 0.0001; ***p < 0.001; **p < 0.01; *p < 0.05; ##p < 0.01; #p < 0.05; ns p > 0.05

We transfected cultured hippocampal neurons with a construct driving expression of hrGFP (humanized *Renilla reniformis* GFP) to visualize the entire neuronal structure with or without a construct encoding for VEGFD—tagged with HA—to achieve VEGFD overexpression (Fig. 3a). 24 h after Bic treatment, neurons expressing only GFP displayed, as expected, an increase in their dendritic length and complexity (Fig. 3b–f). However, neurons additionally expressing VEGFD-HA displayed no activity-induced dendritic growth (Fig. 3b–f). Under these experimental settings, VEGFD-HA expression preceded Bic treatment; thus, to rule out possible effects due to increased levels of VEGFD prior synaptic activity boost, we employed the inducible ESARE (enhanced synaptic activity-regulated element) system. ESARE is a synthetic neuronal activity-dependent promoter that drives gene expression upon synaptic activity [31, 32], thus inducing VEGFD-HA or LacZ-HA expression upon Bic treatment (Fig. 3g, h; Suppl. Fig. 1a–c). In addition, the ESARE vectors ensure the constitutive expression of tDimer as a marker of successful vector delivery and expression in neurons (Fig. 3g; Suppl. Fig. 1a, b). 24 h Bic treatment induced cFos expression in neurons infected with ESARE > LacZ or ESARE > VEGFD, as a result of the increased neuronal activity (Suppl. Fig. 1b), and reduced *VEGFD* mRNA expression in the LacZ control, as expected (Suppl. Fig. 1c). In ESARE > VEGFD expressing neurons, 24 h Bic treatment induced expression of VEGFD at mRNA and protein levels which compensate the activity-triggered decrease of endogenous VEGFD (Suppl. Fig. 1b, c).

Bic-treated neurons expressing ESARE > LacZ had a significant increase in dendritic arborization compared to untreated ESARE > LacZ-expressing neurons (Fig. 3i–l). In contrast, neurons transfected with ESARE > VEGFD, which promotes expression of exogenous VEGFD in response to Bic-stimulation, did not exhibit such an increase (Fig. 3i–l).

As VEGFD is a secreted factor, we tested whether administering rVEGFD in the medium of cultured neurons would also be sufficient to prevent activity-induced structural changes. First, we confirmed that rVEGFD administration activates VEGFR3 by measuring its phospho-tyrosine levels via proximity ligation assay (PLA, Suppl. Fig. 1d). Co-administration of Bic and rVEGFD blunted the Bic-mediated increase in dendritic arborization (Fig. 3m–q) in a similar way to what we observed when we manipulated neurons to overexpress VEGFD (Fig. 3a–l). We checked for possible biological effects due to contaminants which may be present in rVEGFD by repeating the same experiment using rVEGFD produced and purified by a different company (rVEGFD-Alt) and thus likely to have different contaminants. The results were undistinguishable from the ones obtained with rVEGFD and rVEGFD-Alt prevented Bic-induced dendritic remodeling (Suppl. Fig. 1e–g).

Although human VEGFD binds both VEGFR2 and VEGFR3, it was shown that murine VEGFD only binds and activates VEGFR3 [57]. Indeed, our past work indicates that VEGFD acts in murine neurons via VEGFR3 [20, 23, 24]. To define which of the two receptors could be mediating VEGFD’s stabilization effect, we made use of DC101, a neutralizing antibody against VEGFR2 [58]. Preventing VEGFR2 activation via DC101 treatment had no effect either on Bic-induced dendritic growth or on VEGFD’s capacity to prevent remodeling (Suppl. Fig. 1h). Further, in a competition experiment in which we co-delivered rVEGFA—a ligand for VEGFR2—and rVEGFD, the latter still successfully counteracted Bic-triggered dendritic remodeling (Suppl. Fig. 1i). Finally, pharmacological blockade of VEGFR3 via SAR131675 [59] abolished the negative effects that VEGFD has on dendritic structural remodeling (Suppl. Fig. 1j).

Next, we tested whether VEGFC, the closest homologue of VEGFD and also capable to bind VEGFR3, may have similar properties in opposing activity-dependent structural remodeling (Suppl. Fig. 1k–n). Bic-treated neurons increased the size and complexity of their dendritic trees, a process which took also place when we co-administered recombinant VEGFC (rVEGFC). This finding further strengthens the concept that in the nervous system the two factors have distinct functions and VEGFD appears to be the one governing dendrite architecture via VEGFR3 [20, 22, 23].

We complemented the endpoint-based morphometric analyses performed after sample fixation (Fig. 3a–q; Suppl. Fig. 1e–j) with automated, continuous, non-invasive imaging and measurement of neurites of cultured neurons expressing GFP (Fig. 3r, s). Bic-treated cultured neurons displayed a time-dependent increase in neurite length [60] while neurons treated with Bic and rVEGFD did not (Fig. 3t). In agreement with our previous observations (Suppl. Fig. 1k–n), rVEGFC had no effect on Bic-induced neurite outgrowth (Suppl. Figure 1o, p). Our approach to promote structural remodeling relied on Bic to boost synaptic activity. To exclude the possibility that the VEGFD-mediated brake on structural rearrangement could be due to a non-direct effect of VEGFD on dendrites through a possible interference with Bic-triggered activity, we monitored nuclear calcium transients as a proxy for assessing neuronal activity [61]. Bic stimulation evoked repetitive, nuclear calcium rises consistent with action potential bursting [62]; which were still present and similar both in frequency and amplitude when rVEGFD was also applied to the cultures (Suppl. Fig. 2). These data therefore show that VEGFD directly influences dendrite architecture without interfering with neuronal activity.

Taken together, our findings indicate that a reduction of VEGFD level is necessary to allow synaptic activity-dependent structural remodeling.

### VEGFD modifies dendritic dynamics induced by synaptic activity

**Fig. 4:**
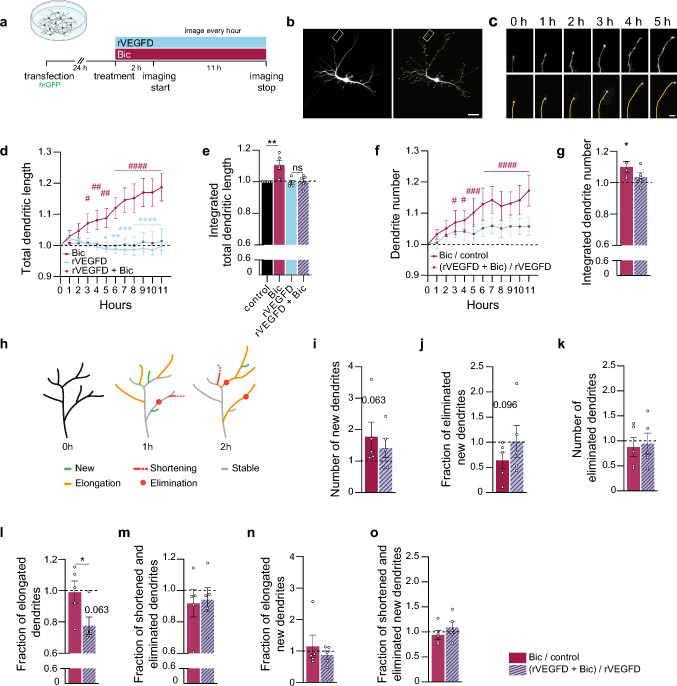
VEGFD modifies synaptic activity-induced dendrite dynamics. **a** Schema of time-lapse imaging experiment of hrGFP-transfected cultured hippocampal neurons, with or without rVEGFD, or bicuculline (Bic) treatment, or both. **b** Representative images of an hrGFP-expressing neuron. An overlay of dendrite traces is shown in the right image. Scale bar = 50 µm. **c** Tracking of an individual dendrite from neuron in (**b**) (marked with a rectangle) at indicated timepoints. An overlay of dendrite traces is shown in the lower row. Scale bar = 10 µm. **d** Total dendritic length over time normalized on the first timepoint and untreated control. **e** Integration of data shown in (**d**). **f** Total dendrite number over time normalized on the first timepoint and respective untreated control. **g** Integration of data shown in (**f**). **h** Representative drawing of dendrite dynamics with branches colored according to their dynamics. Green: new dendrite, orange: elongated dendrite, red: shortened dendrite, red circle: eliminated dendrite, grey: stable dendrite. **i** Total number of new dendrites normalized on respective untreated control. **j** Fraction of new dendrites that were then eliminated normalized on respective untreated control. **k** Total number of eliminated dendrites normalized on respective untreated control. **l** Fraction of elongated dendrites over time normalized on respective untreated control. **m** Fraction of shortened and eliminated dendrites over time normalized on respective untreated control. **n** Fraction of elongated new dendrites over time normalized on respective untreated control. **o** Fraction of shortened and eliminated new dendrites over time normalized on respective untreated control. Two-way ANOVA followed by Dunnett’s post hoc test for comparisons to basal values (**d**, **f**), or Tukey’s (**d**) or Bonferroni’s post (**f**) hoc test for comparisons between conditions; One-way ANOVA followed by Tukey’s post hoc test (**e**); one sample t-test (**g**, **j**, **k**, **m**, **o**) and Wilcoxon signed-rank test (Bic/control in (**i**) and (**n**); (rVEGFD + Bic)/rVEGFD in (**l**)) for comparisons to respective untreated control; or two-tailed unpaired Student’s t-test (**g, j, k, m, o**) or Mann–Whitney test (**i, l, n**) for comparisons between conditions. N = 5 independent culture preparations, 18–23 neurons/condition in total. Graphs represent mean ± SEM. Dots represent single values. Asterisks (*) refer to statistical comparisons between conditions and hashtags (#) to comparisons to basal values per condition. ****p < 0.0001; ***p < 0.001; **p < 0.01; *p < 0.05; ns p > 0.05; ####p < 0.0001; ###p < 0.001; ##p < 0.01; #p < 0.05

We established time-lapse imaging of visually isolated neurons by means of GFP expression combined with machine learning-based reconstruction of the dendritic arbor and tracking of single dendrites. We selected this strategy to determine whether VEGFD-mediated inhibition of structural remodeling was caused by limiting growth or by promoting the destabilization of recently formed dendrites (Fig. 4a–c, h). Initially, we calculated the total dendritic length over time, confirming our earlier findings that VEGFD prevents a time-dependent increase in the total dendritic length that is induced by increasing synaptic activity (via Bic) (Fig. 3; Fig. 4d, e). Subsequently, we calculated the number of dendrites per neuron and found that Bic gradually increases the number of dendrites over time (Fig. 4f, g). Co-administration of rVEGFD reduced, but not fully abolished the activity-induced increase in dendrite number (Fig. 4f, g). We specifically analyzed the subpopulations of newly formed dendrites. Albeit not statistically significant, we observed a consistent formation of new dendrites (Fig. 4i) and a reduction in their elimination following Bic treatment (Fig. 4j). Elimination of the global dendrite population was unchanged (Fig. 4k). Co-treatment with rVEGFD shows a similar pattern regarding the formation of new dendrites (Fig. 4i) and overall number of eliminations (Fig. 4k), although a trend toward destabilization of newly formed dendrites appeared (Fig. 4j). We then categorized dendrites into elongated, shortened—which includes dendrite eliminations—or stable (Fig. 4h, l–o). Both the shortening and elimination of all dendrites (Fig. 4m) and of the subpopulation of newly formed dendrites (Fig. 4o) were not affected by Bic or Bic/rVEGFD. However, co-application of rVEGFD decreased the number of elongated dendrites (Fig. 4l) while the newly formed dendrites showed no difference compared to the Bic-treated ones (Fig. 4n). In sum, these data indicate that VEGFD neither affects the formation of new dendrites nor their elongation and shortening but possibly enhances their destabilization and hinders the synaptic activity-driven elongation of already existing dendrites. In this manner, VEGFD compensates for the synaptic-activity-induced formation of new dendrites resulting in a zero net growth of total dendrite length.

### Fear memory-dependent dendritic remodeling in the adult hippocampus requires reduction of VEGFD expression

**Fig. 5:**
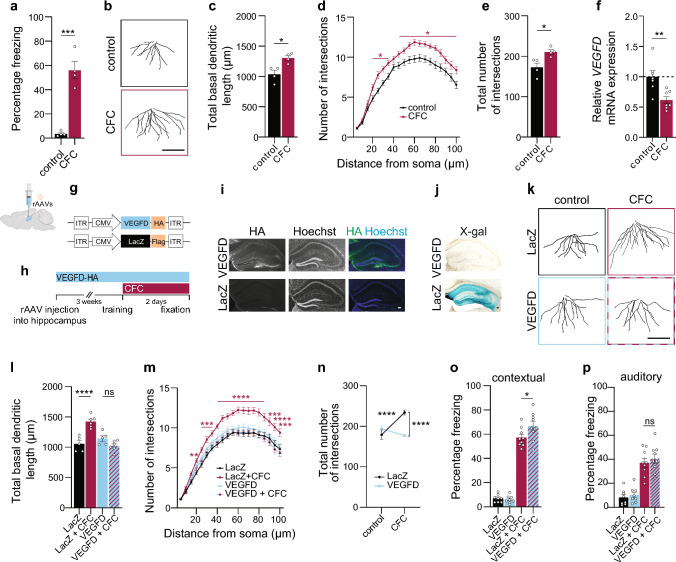
In vivo synaptic activity-dependent structural remodeling is enabled by reduction of VEGFD expression. **a–f** Mice were subjected to contextual fear conditioning (CFC) or not. **a** Percentage of time spent immobile during the contextual memory test N = 4 mice. **b** Representative Golgi tracings of basal dendrites of CA1 pyramidal neurons. Scale bar = 100 µm. **c–e** Total basal dendritic length (**c**), Sholl analysis (**d**) and total number of intersections (**e**) of CA1 pyramidal neurons. Two-tailed unpaired Student’s t-test (**a, c, e**) or multiple unpaired t-test (minimal significant p values are displayed) (**d**). N = 4 mice, 23–24 neurons/condition in total. **f** QRT-PCR analysis of *VEGFD* mRNA expression in the CA1. Two-tailed unpaired Student’s t-test. N = 7 mice. **g**–**p** Mice stereotaxically-injected with rAAV-VEGFD-HA or rAAV-LacZ-Flag were subjected to CFC or not. **g** Schema of rAAV constructs used. **h** Schema of the experimental setup. **i** Representative images of HA-immunostained dorsal hippocampi expressing VEGFD-HA or LacZ-Flag. Nuclei were labelled with Hoechst. Scale bar = 100 µm.** j** Representative images of X-gal-stained dorsal hippocampi expressing LacZ-Flag or VEGFD-HA. Scale bar = 100 µm. **k** Representative Golgi tracings of basal dendrites of CA1 pyramidal neurons. Scale bar = 100 µm. **l**–**n** Total basal dendritic length (**l**), Sholl analysis (**m**) and total number of intersections (**n**) of CA1 pyramidal neurons. One- and Two-way ANOVA followed by Bonferroni’s post hoc test (**l**, **n**), or Dunnett’s post hoc test (**m**). N = 5–6 mice, 23–30 neurons/condition in total. **o** Percentage of time spent immobile during the contextual memory test N = 8–9 mice. **p** Percentage of time spent immobile during the played tone for the cued auditory memory test. N = 8–9 mice. One-way ANOVA followed by Bonferroni’s post hoc test (**o**, **p**). Graphs represent mean ± SEM. Dots represent single values. ****p < 0.0001; ***p < 0.001; **p < 0.01; *p < 0.05; ns p > 0.05

In cultured neurons, dendritic remodeling involved a reduction in VEGFD levels (Figs. 3 and 4); therefore, we investigated whether this observation would be valid in vivo in adult mice. Induction of fear memory in adult mice increases both the length and complexity of dendrites of CA1 hippocampal neurons [10]. We thus decided to employ a similar approach and subject mice to contextual and cued fear conditioning (CFC) to promote dendrite remodeling of hippocampal neurons in vivo. First, we confirmed that CFC established fear memory and caused an increase in length and complexity of the basal CA1 dendrites (Fig. 5a–e). Further, we detected lower *VEGFD* mRNA levels in the isolated CA1 region of the mice subjected to CFC in comparison to control mice (Fig. 5f). Next, we employed stereotaxic surgery to deliver rAAVs driving expression of VEGFD-HA or LacZ into the adult dorsal hippocampus of mice to test whether maintaining high VEGFD levels affects CFC-induced dendritic growth (Fig. 5g, h). Three weeks after delivery of rAAVs, we confirmed expression of the employed rAAVs (Fig. 5i, j). When we subjected the rAAV-injected mice to CFC, CA1 neurons of LacZ-expressing mice still displayed an induction of basal dendritic length and complexity after CFC (Fig. 5k–n). On the contrary, the dendritic structure of CA1 neurons of rAAV-VEGFD-injected mice remained unchanged (Fig. 5k–n). Both LacZ- and VEGFD-expressing mice displayed strong freezing responses after CFC. (Fig. 5o). Surprisingly, VEGFD-expressing mice, in which we interfered with the activity-dependent downregulation of VEGFD and subsequent dendritic remodeling (Fig. 5k–n), showed aberrantly increased freezing behavior (Fig. 5o). Additionally, we assessed auditory cued fear memory that relies less on the function of the hippocampus [63]. Both LacZ-and VEGFD-expressing mice learned the negative association to the tone (cue) and had similar freezing responses (Fig. 5p). Overall, these findings demonstrate that a decrease in VEGFD expression is necessary for synaptic activity-dependent structural remodeling in vivo and suggest a role for hippocampal dendrite remodeling as a regulatory mechanism in correct memory formation.

### VEGFD induces dephosphorylation of ezrin on Y478 residue via STEP activation

**Fig. 6:**
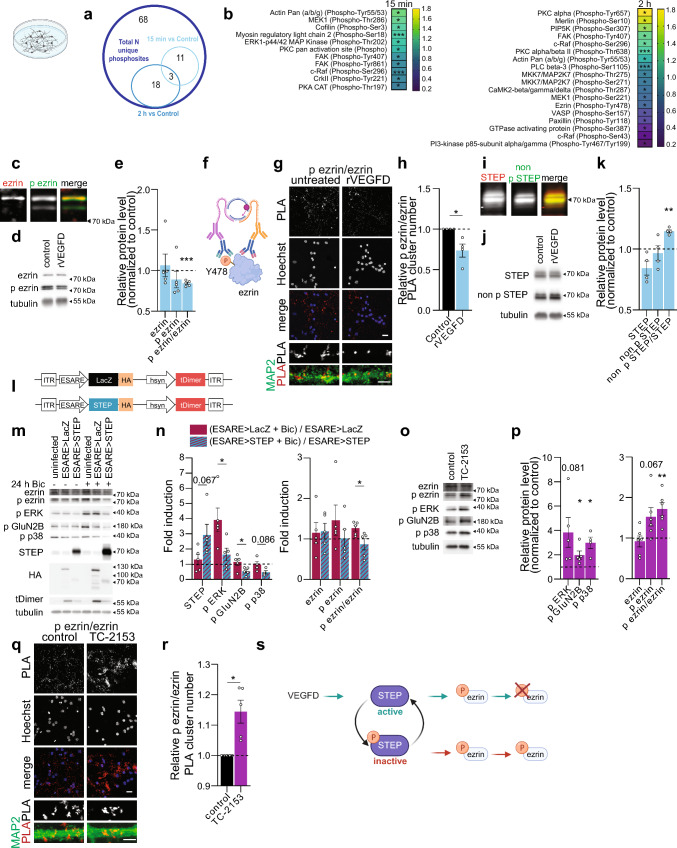
VEGFD activates the tyrosine phosphatase STEP and promotes dephosphorylation of ezrin. **a**, **b** Number (**a**) and list (**b**) of cytoskeleton-associated proteins showing significant change in their phosphorylation (up or down) 15 min or 2 h after rVEGFD treatment normalized on untreated control. Multiple paired t-test. N = 3 independent culture preparations. **c** Western blot using two-color infrared fluorescent protein detection of total ezrin (red) and phospho-ezrin (green) with lysate from cultured hippocampal neurons confirming that the upper band corresponds to ezrin. **d**, **e** Immunoblotting of cultured hippocampal neurons with or without rVEGFD treatment for 2 h. **d** Representative immunoblots of total ezrin, phospho-Y478 ezrin, and tubulin. **e** Total ezrin and phospho-Y478 ezrin and their phospho/total ratio normalized to tubulin expression and control. One sample t-test (phospho/total ratio) and Wilcoxon signed-rank test (ezrin and phospho-ezrin). N = 5 independent culture preparations. **f**–**h** Proximity ligation assay (PLA) with antibodies against ezrin and phospho-Y478 ezrin of cultured hippocampal neurons with or without rVEGFD treatment for 2 h. **f** Schema of PLA for detection of phospho-proteins. **g** Representative images of PLA signal in neurons treated as indicated. Nuclei were labelled with Hoechst. Scale bar = 20 µm. Lower panels show higher magnification of dendrites labeled with MAP2. Scale bar = 5 µm. **h** PLA cluster number normalized on the number of Hoechst-labelled cells and on control. One sample t-test. N = 4 independent culture preparations. **i** Western blot using two-color infrared fluorescent protein detection of total STEP (red) and non-phospho-S221 STEP (green) with lysate from cultured hippocampal neurons, confirming that both bands correspond to STEP. **j, k** Immunoblotting of cultured hippocampal neurons with or without rVEGFD for 2 h. **j** Representative immunoblots of total STEP, non-phospho-S221 STEP, and tubulin. **k** Total STEP and non-phospho-S221 STEP and their phospho/total ratio normalized to tubulin expression and control. One sample t-test. N = 4 independent culture preparations. **l**–**n** Immunoblot analysis of cultured hippocampal neurons infected with rAAV-ESARE > STEP-HA or rAAV-ESARE > LacZ-HA, with or without bicuculline (Bic) treatment to activate the ESARE promoter. **l** Schema of the rAAVs constructs used. **m** Representative immunoblots of total ezrin, phospho-Y478 ezrin, phospho-ERK, phospho-GluN2B, phospho-p38, STEP, HA, tDimer, and tubulin. **n** STEP, phospho-ERK, phospho-GluN2B, phospho-p38, total ezrin, phospho-Y478 ezrin, and phospho-Y478/total ezrin normalized to tubulin expression and respective baseline condition. Two-tailed unpaired Student’s t-test. N = 4-5 independent culture preparations. **o**, **p** Immunoblotting of cultured hippocampal neurons treated with TC-2153 or DMSO (vector) for 1 h. **o** Representative immunoblots of total ezrin, phospho-Y478 ezrin, phospho-ERK, phospho-GluN2B, phospho-p38 and tubulin. **p** Phospho-ERK, phospho-GluN2B, phospho-p38, total ezrin, phospho-Y478 ezrin and phospho-Y478/total ezrin normalized to tubulin expression and control. One sample t-test. N = 4–5 independent culture preparations. **q**, **r** PLA with antibodies against ezrin and phospho-Y478ezrin of cultured hippocampal neurons treated with TC-2153 or DMSO (vector) for 1 h. **q** Representative confocal images of PLA signal in neurons treated as indicated. Nuclei were labelled with Hoechst. Scale bar = 20 µm. Lower panels show higher magnification of dendrites labeled with MAP2. Scale bar = 5 µm **r** PLA cluster number normalized on the number of Hoechst-labelled cells and on control. One sample t-test. N = 5 independent culture preparations. **s** Schema of VEGFD-mediated dephosphorylation of ezrin. VEGFD signaling leads to the dephosphorylation of STEP. Dephosphorylated S221 STEP is active and dephosphorylates Y478 ezrin. Graphs represent mean ± SEM. Dots represent single values. ***p < 0.001; **p < 0.01; *p < 0.05

Prompted by our results showing that VEGFD influences the structure of neurons and elements of the cytoskeleton (Figs. 1, 2, 3, 4, 5), we performed a phospho-antibody array of proteins associated with cytoskeleton regulation to identify the proteins that VEGFD affects in neurons. We treated hippocampal neurons for 15 min or 2 h with rVEGFD and the homogenates were probed using a glass-based phospho-antibody assay based on a total array of 143 different antibodies sampling 68 distinct phosphosites and the total expression level of 55 proteins. Our analysis revealed a list of proteins whose phosphorylation was increased or decreased because of rVEGFD administration (Fig. 6a, b).

Among those, because VEGFD modulates cellular stiffness (Fig. 1c) [47], our attention was drawn to ezrin and its phosphosite tyrosine 478. This phosphosite is also referred to as tyrosine 477, which corresponds to the site in chicken where ezrin was initially purified [64]. Ezrin serves as a physical linker between the cortical actin cytoskeleton and the plasma membrane playing a vital role in regulating membrane tension [65]. Ezrin additionally binds and regulates MT organization at the cell cortex [66, 67]. An impairment in ezrin phosphorylation at tyrosine 478 reduces the formation of actin-rich protrusions in epithelial cancer cells [33, 68] and tumor-induced endothelial branching [69]. We confirmed that rVEGFD reduced the levels of pY478 ezrin in neurons using both immunoblotting (Fig. 6c–e) and PLA (Fig. 6f–h).

Src has been shown to phosphorylate Y478 ezrin in human keratinocytes, mouse fibroblasts and HEK 293 cells [68, 70, 71]. To test whether this was also the case in neurons, we treated neurons with PP2, a known inhibitor of Src family kinases [68, 70, 71], for different periods of time (Suppl. Fig. 3a, b). As expected, phosphorylation of Akt (pAkt), which is regulated by Src through the PI3K/AKT pathway, was reduced in PP2-treated neurons (Suppl. Fig. 3a, b) [72]. However, pY478 ezrin was not affected by PP2 treatment (Suppl. Fig. 3a–d). Furthermore, we re-checked the values of the phospho-antibody array, as it also included four different antibodies that detect Src phospho-sites. rVEGFD treatment, at either 15 min or 2 h time point, did not affect any of the four Src phospho-sites (Suppl. Fig. 3e). Finally, we directly assessed pSrc levels in response to rVEGFD and found no significant changes. (Suppl. Fig. 3f–i). These data seem thus to exclude Src as possible kinase in the regulation of ezrin Y478 phosphorylation in neurons.

Next, we shifted our attention to the possible phosphatase modulating dephosphorylation of Y478 ezrin. The identity of the phosphatase acting on Y478 ezrin is not clear. Recently, ezrin was identified in the interactome of the striatal-enriched protein tyrosine phosphatase (STEP) [73]. STEP is a brain-specific protein phosphatase that regulates a variety of functions, including dendrite and spine morphology [74–76]. Thus, we tested whether STEP may be responsible for the VEGFD-mediated dephosphorylation of Y478 ezrin. We treated neurons with rVEGFD and then assessed the activation state of STEP via immunoblotting using an antibody that detects only the non-phosphorylated (S221) form of STEP that indicate more activated STEP (Fig. 6i–k, s) [77, 78]. rVEGFD administration significantly increased non phosphorylated/active STEP (Fig. 6i–k) supporting the hypothesis that STEP may dephosphorylate Y478 ezrin in response to VEGFD.

Since higher STEP expression is associated with many neurodegenerative diseases [74, 76] and overexpression of STEP leads to memory impairments in mice, we decided to use the inducible ESARE system to reduce the time window of exposure of neurons to high STEP levels (Fig. 6l). Upon triggering the ESARE-driven overexpression of STEP, we detected a significant decrease in the phosphorylation state of the known STEP targets ERK, GluN2B and p38 [77, 79] (Fig. 6m, n). Further, inducing expression of STEP was sufficient to lower phosphorylation of Y478 ezrin (Fig. 6m, n). Next, we pharmacologically inhibited STEP activity with TC-2153 [75, 77, 79]. As expected, STEP inhibition caused an increase in the phosphorylation level of known STEP targets (Fig. 6o, p). In addition, inhibition of STEP resulted in higher levels of pY478 ezrin (Fig. 6o–r). STEP activation can be modulated by calcineurin [80, 81], which is also known to activate NFAT by dephosphorylating multiple residues in its regulatory domain [82]. We observed a reduction in the levels of pNFATc1 upon rVEGFD treatment (Suppl. Fig. 3j, k). Moreover, we detected an increase of phosphate released from a calcineurin-specific substrate—and thus indicative of calcineurin activity—following rVEGFD treatment (Suppl. Fig. 3l).

Taken together, these data suggest that VEGFD modulates the phosphorylation state of ezrin on Y478 residue by regulating STEP activation possibly via calcineurin (Fig. 6s).

### The phosphomutant Y478F ezrin inhibits synaptic activity-dependent remodeling in vitro

**Fig. 7:**
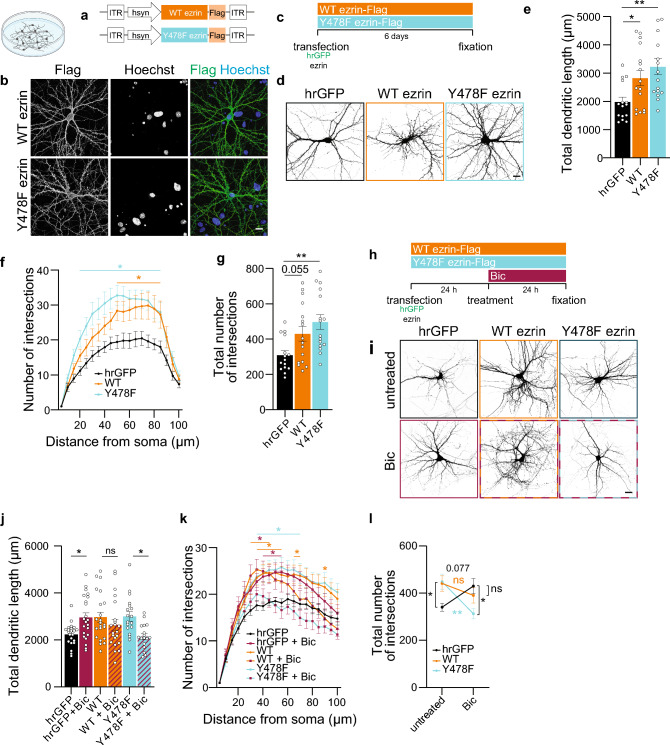
Y478 ezrin phosphorylation modulates synaptic activity-dependent remodeling in cultured neurons. **a**–**g** Morphometric analyses of cultured hippocampal neurons transfected with hrGFP (vector) and/or WT ezrin-Flag or Y478F ezrin-Flag. **a** Schema of the constructs used. **b** Representative images of WT or Y478F ezrin-expressing neurons. Flag-tag was detected immunocytochemically. Nuclei were labelled with Hoechst. Scale bar = 20 µm. **c** Schema of the experimental setup. **d** Representative images of neurons transfected and treated as indicated. hrGFP was used to visualize neurons. Scale bar = 20 µm. **e**–**g** Total dendritic length (**e**), Sholl analysis (minimal significant p values are displayed) (**f**) and total number of intersections (**g**) of neurons transfected and treated as indicated. One-way (**e, g**) or Two-way ANOVA followed by Dunnett’s post hoc test (**f**). N = 15–16 neurons from 3 independent culture preparations. **h**–**l** Morphological analysis of cultured hippocampal neurons transfected with hrGFP (vector) and/or WT ezrin-Flag or Y478F ezrin-Flag, with or without bicuculline (Bic) treatment for 24 h. **h** Schema of the experimental setup. **i** Representative images of neurons treated as indicated. hrGFP was used to visualize neurons. Scale bar = 20 µm. **j**–**l** Total dendritic length (**j**), Sholl analysis (minimal significant p values are displayed) (**k**) and total number of intersections (**l**) of neurons treated as indicated. One-way and Two-way ANOVA followed by Bonferroni’s or Tukey’s post hoc test (**j**, **l**), or Dunnett’s post hoc test (**k**). N = 17–22 neurons from 5 independent culture preparations. Graphs represent mean ± SEM. Dots represent single values. **p < 0.01; *p < 0.05; ns p > 0.05

VEGFD hinders structural changes of dendrites, modulates cytoskeleton elements and the phosphorylation state of the cytoskeleton-related protein ezrin. We therefore explored the possible impact of ezrin on dendritic architecture. To this end, we generated constructs to achieve overexpression of WT ezrin or of a mutant form of ezrin which cannot be phosphorylated at Y478 residue as the tyrosine has been substituted with a phenylalanine (Y478F; Fig. 7a). Both constructs were successfully expressed and distributed in the entire neuron (Fig. 7b, c). Overexpression of WT or Y478F mutant ezrin increased dendritic length and arborization (Fig. 7a–g) and impacted expression of both *VEGFD* and *VEGFR3* (Suppl. Fig. 4).

We next induced dendritic structural plasticity via Bic treatment (Figs. 3, 4; Suppl. Fig. 1e–p). As expected, vector-expressing neurons had a more complex dendritic tree after Bic treatment; however, neurons expressing WT ezrin, which already had a greater dendritic arborization at resting conditions, failed to further extend their dendritic tree, possibly due to reaching growth limitation (Fig. 7h–l). Surprisingly, neurons expressing the phospho-deficient mutant Y478F ezrin displayed an opposite phenotype, and their dendrites not only did not grow, but rather reverted to control values (Fig. 7h–l). Thus, increased levels of ezrin promote dendritic growth and the phosphorylation state of residue Y478 determines neuronal response to remodeling inputs.

### Phosphomutant Y478F ezrin modifies synaptic activity-induced dendrite dynamics

**Fig. 8:**
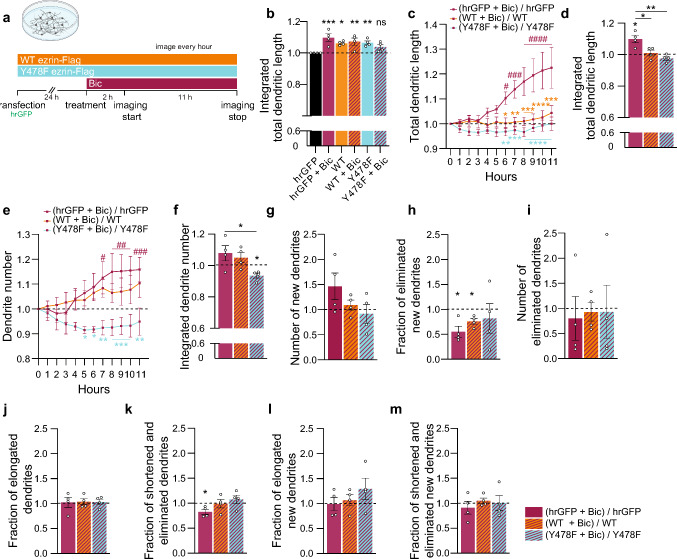
Y478F ezrin alters synaptic activity-induced dendrite dynamics. **a** Schema of time-lapse imaging experiment of hrGFP (vector) and/or WT ezrin-Flag or Y478F ezrin-Flag-transfected cultured hippocampal neurons with or without bicuculline (Bic) treatment.** b** Integration of total dendritic length over time normalized on the first timepoint and untreated control. **c** Total dendritic length over time normalized on the first timepoint and respective untreated control. **d** Integration of data shown in (**c**). **e** Total dendrite number over time normalized on the first timepoint and respective untreated control. **f** Integration of data shown in (**e**). **g** Total number of new dendrites normalized on respective untreated control. **h** Fraction of new dendrites that were eliminated normalized on respective untreated control.** i** Total number of eliminated dendrites normalized on respective untreated control. **j** Fraction of elongated dendrites over time normalized on respective untreated control. **k** Fraction of shortened and eliminated dendrites over time normalized on respective untreated control. **l** Fraction of elongated new dendrites over time normalized on respective untreated control. **m** Fraction of shortened and eliminated new dendrites over time normalized on respective untreated control. Two-way ANOVA followed by Dunnett’s post hoc test for comparisons to basal values (**c**, **e**), or Tukey’s post hoc test for comparisons between conditions (**c, e**); One-way ANOVA followed by Dunnett’s post hoc test (**b**) or Tukey’s post hoc test (**d**, **f**–**m**); one sample t-test for comparisons to respective untreated control (**d, f**–**m**). N = 4 independent culture preparations, 13–16 neurons/condition in total. Graphs represent mean ± SEM. Dots represent single values. Asterisks (*) refer to statistical comparisons between conditions and hashtags (#) to comparisons to basal values per condition. ****p < 0.0001; ***p < 0.001; **p < 0.01; *p < 0.05; ns p > 0.05; ####p < 0.0001; ###p < 0.001; ##p < 0.01; #p < 0.05

Since our data indicate that ezrin and the phosphorylation state of its residue Y478 modulate dendritic trees and how they respond to activity, we used single tracking of dendrites with machine learning to characterize the behavior of dendrites in Y478F-ezrin-expressing neurons in response to increased synaptic activity (Fig. 8a).

In agreement with our previous findings (Fig. 7), overexpression of WT or Y478F ezrin is sufficient to increase dendritic total length (Fig. 8b). Moreover, Bic treatment fails to increase total length in neurons expressing WT or Y478F ezrin but nevertheless raises total length in control neurons (Fig. 8b–d). As expected, activity increased the number of dendrites over time in control neurons (Figs. 4f, g, 8e, f). This effect was less pronounced in WT ezrin and surprisingly, in Y478F ezrin-expressing neurons, even decreased in comparison to untreated (Fig. 8e, f). In contrast to control neurons, which exhibit a tendency toward increased formation of new dendrites with Bic (Figs. 4i, 8g), WT and Y478F ezrin-expressing neurons do not (Fig. 8g). In comparison to control or WT ezrin-expressing neurons, for which Bic increased the lifetime of newly formed dendrites by reducing their elimination (Figs. 4j, 8h), Y478F ezrin-expressing neurons had an opposite behavior and Bic failed to stabilize newly formed dendrites (Fig. 8h). Dendrite elimination in general was not impacted (Fig. 8i). Additionally, neither WT nor Y478F ezrin had any effect on the elongation, shortening, or elimination of any dendrites (Fig. 8j, k) or within the subpopulation of recently formed dendrites (Fig. 8l, m).

In sum, in response to synaptic activity, overexpression of WT ezrin renders neurons refractory to further increase their dendrite number and extend their length, while Y478F ezrin reduces dendrite number through destabilization of newly formed dendrites.

### Phosphomutant Y478F ezrin inhibits dendritic remodeling induced by fear memory in the adult hippocampus

**Fig. 9:**
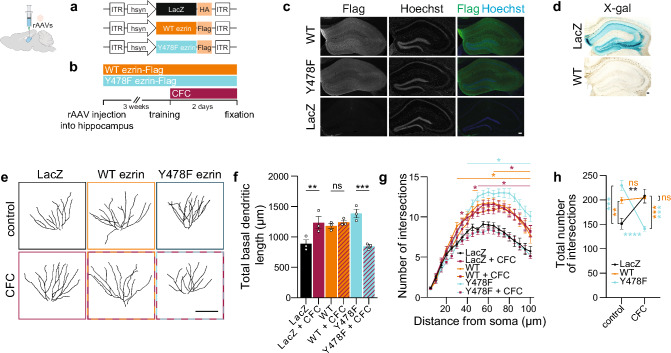
Phospho-mutant Y478F ezrin blocks activity-dependent remodeling in vivo. Mice stereotaxically-injected with rAAV-WT ezrin-Flag, rAAV-Y478F ezrin-Flag or rAAV-LacZ-HA were subjected to contextual and cued fear conditioning (CFC) or not. **a** Schema of rAAV constructs. **b** Schema of the experimental setup. **c** Representative images of Flag-immunostained dorsal hippocampi expressing WT ezrin-Flag, Y478F ezrin-Flag or LacZ-HA. Nuclei were labelled with Hoechst. Scale bar = 100 µm.** d** Representative images of X-gal-stained dorsal hippocampi expressing LacZ-HA or WT ezrin-Flag. Scale bar = 100 µm. **e** Representative Golgi tracings of basal dendrites of CA1 pyramidal neurons. Scale bar = 100 µm. **f**–**h** Total basal dendritic length (**f**), Sholl analysis (minimal significant p values are displayed) (**g**) and total number of intersections (**h**) of CA1 pyramidal neurons. One-way and Two-way ANOVA followed by Bonferroni’s or Tukey’s post hoc test (**f**, **h**), or Dunnett’s post hoc test (**g**). N = 3 mice, 11–15 neurons/condition in total. Graphs represent mean ± SEM. Dots represent single values. ****p < 0.0001; ***p < 0.001; **p < 0.01; *p < 0.05; ns p > 0.05

Lastly, to investigate the effect of manipulating ezrin expression and phosphorylation level on dendrite arborization in vivo*,* we stereotaxically delivered rAAVs driving expression of LacZ, WT or Y478F ezrin into the adult dorsal hippocampus of mice (Fig. 9a, b). Three weeks after delivery of rAAVs, we confirmed expression of the rAAVs (Fig. 9c, d). Similar to cultured neurons (Fig. 7), overexpression of either WT or Y478F ezrin prompted an increase in the length and complexity of dendrites (Fig. 9e–h). Following CFC, the CA1 neurons of LacZ-expressing mice had a higher basal dendritic length and complexity (Figs. 5, 9e–h). WT ezrin-expressing neurons, in agreement with the observations for cultured hippocampal neurons, failed to further extend their dendritic arbor (Fig. 9e–h). Finally, the dendritic structure of CA1 neurons of rAAV-Y478F ezrin-injected mice reverted to control values (Fig. 9e–h).

## Discussion

**Fig. 10:**
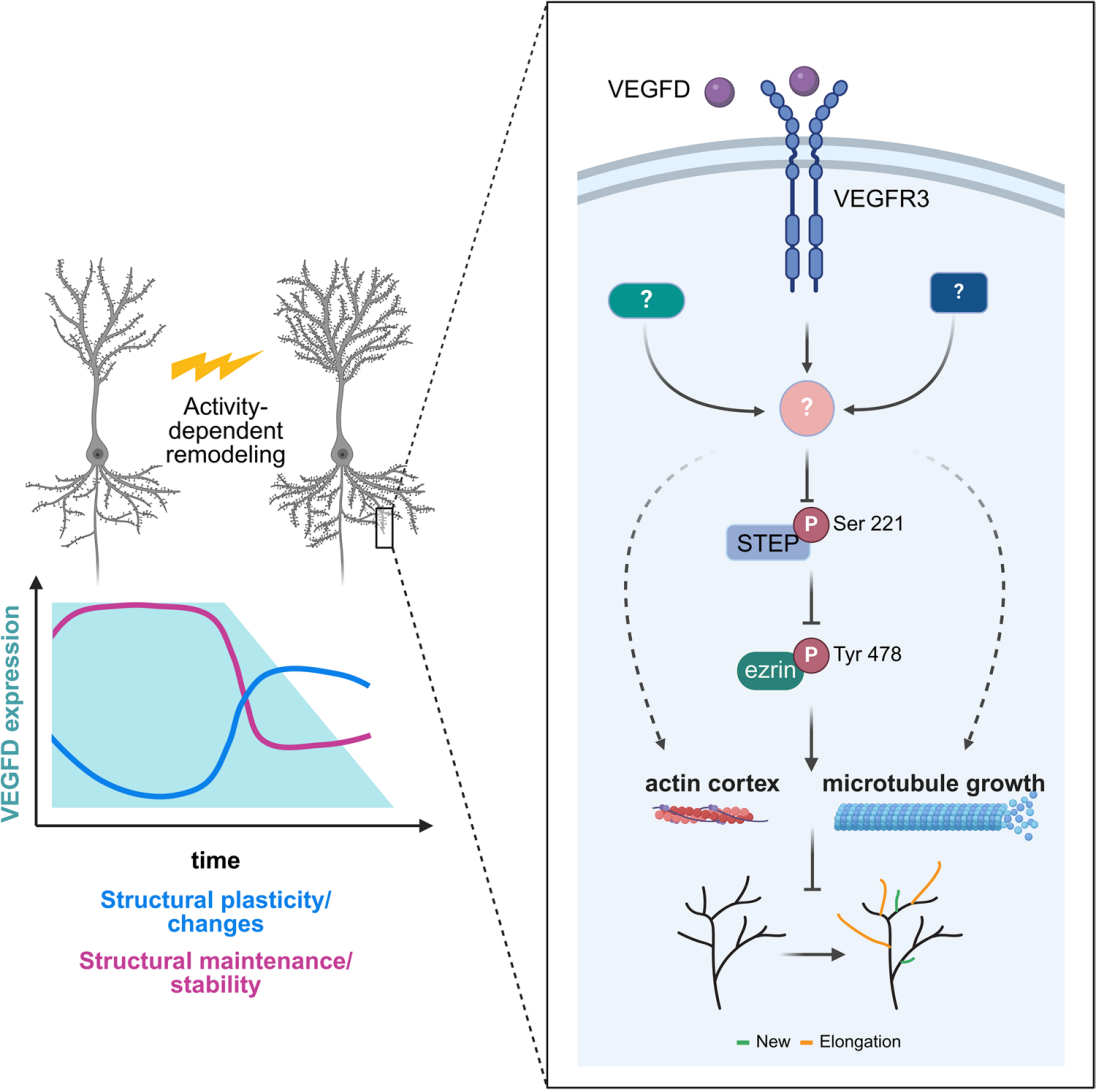
VEGFD signaling balances stability and activity-dependent plasticity of dendrites. VEGFD signaling plays a crucial role in regulating the equilibrium between dendritic maintenance and plasticity. Activity-dependent alterations can take place only when VEGFD expression is downregulated. VEGFD preserves the original morphological state of neurons by influencing the actin cortex and microtubule growth, thereby preventing the elongation of dendrites, and destabilizing newly formed dendrites. VEGFD signaling induces the dephosphorylation of STEP at S221, leading to its activation, and subsequently, STEP dephosphorylates the cytoskeleton-associated protein ezrin at Y478

In this study, we show that VEGFD modulates the cytoskeleton and opposes activity-dependent plasticity of dendrites while promoting their stabilization (Fig. 10). Moreover, we identify the cytoskeleton-associated protein ezrin as a downstream mediator of VEGFD signaling involving activation of STEP.

VEGFD is known for modulating vascular and neuronal morphology [20–24, 40–43]. Here, the impact of VEGFD on cytoskeleton elements in neurons was characterized for the first time and revealed its selective effect on cortical actin and microtubules (MTs). In agreement with previous findings, namely that VEGFD does not modulate spine structure or density [20, 42], our results show that spine actin dynamics, which are the core component defining spine morphology [83], are not affected by VEGFD. Actin is mostly found in spines, but it also plays a part in controlling the entire structure of neurons as it is a component of the contractile actomyosin network. The actin network underneath the cell membrane—also referred to as the actin cell cortex—is critical in regulating membrane tension and regulates cell shape and elasticity [44, 84]. We found that VEGFD leads to an increase of cell membrane rigidity in neurons, consistent with an effect of VEGFD on cortical actin. Indeed, similar observations linking VEGFD to membrane stiffness were reported for different cells [47]. Although our results suggest an effect on cortical actin, we cannot exclude a contribution by MTs as the formation of stable MT bundles may contribute to higher membrane stiffness in neurons [85]. MTs offer physical support to dendrites and axons and exert mechanical force by polymerization to grow into a newly formed or elongating branch [12, 86–88]. We found that VEGFD lengthens the lifetime of EB3 comets and decreases the speed of MT growth. A longer lifespan suggests that MT growth is not continuously halted or MT disassembled (catastrophe), which lowers MT dynamics and boosts MT stability. On the other hand, neurite remodeling, including neurite loss, is linked to enhanced MT dynamics [89–92]. Therefore, one of the processes supporting VEGFD-mediated dendrite maintenance is probably VEGFD-induced MT stabilization.

We observed that signals initiating dendritic remodeling caused VEGFD levels to drop. This phenomenon is surprising, since VEGFD expression is known to be necessary for the maintenance of dendrite morphology in mature neurons [20, 22]. Moreover, under neurodegenerative conditions, the activation of extrasynaptic NMDARs by toxic levels of glutamate causes a rapid loss of VEGFD which in turn causes dendrite damage and atrophy [23, 24]. It therefore may seem difficult to reconcile the findings that a decrease in VEGFD levels could be detrimental in degenerative conditions as well as essential for physiological dendrite remodeling. The kinetics of the decrease of VEGFD levels, however, are remarkably different in the two scenarios. We show here, that prolonged increased synaptic activity is required to detect a significant reduction of VEGFD levels, while ten minutes of toxic stimuli is all that is needed for a dramatic decrease of VEGFD with almost complete VEGFD shutoff within 30 min [23]. Furthermore, synaptic and extrasynaptic NMDAR signaling, which are activated by bicuculline treatment or toxic conditions, respectively, trigger opposing pathways, further explaining the variations in the biological outcomes and in the kinetics of VEGFD downregulation. It is also true that synaptic or extrasynaptic pathways act in different ways on the cytoskeleton and trigger either dendritic growth or atrophy [3, 93, 94]. It thus seems that structural changes, whether dendrite loss or growth, involve a reduction of VEGFD levels. Dendrite loss is caused by the fast loss of VEGFD resulting from a pathological insult, whereas dendrite growth is allowed by a slower, gradual reduction of VEGFD prompted by prolonged bursts of synaptic activity. This dualistic behavior of VEGFD in the regulation of cell morphology is, however, not unique. The RNA-binding protein pumilio, for instance, is such a precedent. While in developing neurons the absence of pumilio is linked to increased growth and branching of dendrites, in mature neurons, reducing pumilio levels is associated with a reduction in dendritic spines and a rise in elongated dendritic filopodia [95]. Semaphorin 3A, which is a chemoattractant for dendrites, but a chemorepellent for axons [96], and VEGFA, another member of the VEGF family, whose inactivation decreases or increases spine density in developing or adult olfactory bulb granule cells, respectively [97] are further examples of the complex regulation of neuronal architecture.

Silencing of VEGFR3 expression in neurons results in the same phenotype as silencing of VEGFD expression and (i.e., shorter dendrites and simplified dendritic arborization) [20]. Intravitreal NMDA injection causing structural damage to retinal neurons decreased VEGFD expression in retinal ganglion cells (RGCs; [24]). VEGFD supplementation via neuronal-specific, viral-mediated expression or acute intravitreal delivery of rVEGFD preserved RGC structure and function [24]. Viral-mediated suppression of expression of VEGFR3 specifically in RGCs revealed that VEGFD exerts its protective capacity directly on RGCs via VEGFR3 [24]. In mice, VEGFD binds and activates VEGFR3 while human VEGFD also acts on VEGFR2 [57]. It was shown that VEGFA-VEGFR2 shape dendritic trees of CA3 neurons in the early phases of development [98] while at the same time having constraint capacity on their axonal development [58]. Here, we surprisingly found that boosting activity leads to a significant decrease in the expression levels of VEGFR2. Moreover, interfering with VEGFR2 activation had no consequences in the VEGFD-mediated stabilization of dendritic architecture which was, instead, impaired when VEGFR3 was inhibited. Thus, neuronal VEGFR3 seems to be primarily responsible for the stabilization of dendrites by VEGFD.

Controlled structural dendrite plasticity is crucial during development and, in adulthood, to facilitate cognitive processes but also adjustments to a changing environment, stress, or injury [9, 10, 99]. Dendrite remodeling can also be pathological, when aberrant morphology due to maladaptive plasticity affects brain function negatively. This is for example the case in epilepsy, where deregulated neuronal activity leads to dendritic sprouting and maladaptive dendrite remodeling is associated with neuropathic pain and addiction [100–102]. Here, we found that interfering with fear memory-induced dendritic remodeling by preventing VEGFD downregulation unexpectedly led to stronger freezing responses, indicating increased spatial memory. There are many examples of memory being enhanced by various types of manipulation. If the enhancement is in response to facilitation of a particular cellular process, then that process is interpreted as a positive regulator of memory. If the cellular process is inhibited, it may instead be a mechanism that constrains memory formation. Preventing VEGFD downregulation enhances memory in contextual fear conditioning thus falls into the second category and suggests a role for dendritic remodeling as a possible constrain mechanism. The number of reported memory suppressors supports the idea of a biological need to limit memory formation, and indeed there are several hypotheses to justify the existence of memory suppression mechanisms [103]. Our findings now open a fascinating new chapter in this dynamic scenario and could spur more research into the connection between memory processes and dendritic remodeling. The mechanisms responsible for establishing and preserving a certain, ideal morphological set point are therefore crucial for governing how the nervous system operates. It is noteworthy that VEGFD levels are known to be low during the period of time that is associated with dendritogenesis [20]. Further, pathological loss of VEGFD leads to dendrite atrophy [20, 23, 24], and here we demonstrate that failure to actively downregulate VEGFD prevents physiological dendrite remodeling associated to memory formation. Our thorough examination of VEGFD’s effects on activity-driven dendritogenesis also showed that VEGFD may function in a homeostatic manner by preventing elongation of dendrites and destabilizing newly formed dendrites, thus promoting the maintenance of the original morphological status of neurons. Taken together, all this evidence suggests that VEGFD levels must be low to allow any sort of structural changes and, therefore, VEGFD acts as a molecular brake opposing alterations of dendritic architecture.

We found multiple VEGFD-modulated proteins linked to the actin cortex, which, together with the findings that VEGFD increases the neuronal cortical stiffness, support the idea that VEGFD may act on the cell cortex. Among those proteins, we revealed that VEGFD specifically promotes the dephosphorylation—and consequently the activation—of STEP at S221 [78], possibly involving calcineurin activation; this results in the dephosphorylation of ezrin at Y478. Our data seem to exclude Src as the putative kinase phosphorylating this residue in neurons under modulation by VEGFD.

Interestingly, it was demonstrated that Src contributes to the VEGF/VEGFR2 signaling which is key to the early developmental stages of dendrites and axons of CA3 neurons [58, 98]. These molecular actors are not involved in the VEGFD/VEGFR3-mediated stabilization of mature dendrites and the identity of the kinase phosphorylating Y478 ezrin in this context remains elusive. Our phospho-screening additionally detected a reduction in the phosphorylation of protein kinase A (PKA), indicative of lower PKA activity [104]. Given that PKA is a known regulator of pS221 STEP [78], this may represent an additional passage in the VEGFD signaling cascade. Moreover, calcineurin/ dopamine/adenosine-3′,5′-monophosphate-regulated phosphoprotein 32 (DARPP-32)/ protein phosphatase 1 (PP1) also regulate pS221 STEP [80, 81, 105]. Furthermore, STEP and ezrin may be initially recruited shortly after VEGFD-mediated input and thus represent the *primum movens* in the VEGFD signaling cascade, while the long-term changes in dendritic stabilization may involve other yet unidentified players. The additional and specific elements in the VEGFD signaling cascade as well as their temporal regulation and impact remain to be defined and represent a dynamic field of study to pursue in the future. On this line, it is important to highlight that in this study we made use of a cytoskeleton-focused phospho-antibody array and that a different unbiased approach, such as Mass Spectrometry, may reveal additional key players.

Ezrin is a mediator of developmental neuritogenesis, filopodia formation and axonal growth [106–108]. Recently, ezrin was linked to urokinase-type plasminogen activator-induced dendrite branching [109]. In line with these findings, we detected an ezrin-promoted increase in dendrite length and complexity of neurons both in vitro and in vivo. Our data further revealed that the phosphorylation state of Y478 ezrin governs structural stability during activity-dependent dendritogenesis. Indeed, Y478F impairs the formation of actin-rich protrusions in epithelial and endothelial cells in the context of tumor-induced invasion and of angio-/ and lymphangiogenesis [33, 68, 69]. On the other hand, Y478F-expressing keratinocytes displayed increased myosin light chain bundles, indicative of increased cell protrusions [70]. The mechanisms behind p478 ezrin-mediated effects on cell morphology are not well understood. As a membrane-actin-linker, ezrin modulates cortical actin anchorage and membrane tension [65]. Recently, it was proposed that formation of cellular protrusions may require local detachment of cortical actin from the plasma membrane to release tension mediated by local reductions of ezrin [110]. In addition, ezrin may serve as a docking site for other cytoskeleton-associated proteins or signaling molecules acting on the cytoskeleton [33, 111]. For instance, pY478 ezrin is involved in the regulation of the mammalian target of rapamycin (mTOR) targets 4E-BP1 and p70S6K [68], which play a role in dendrite arborization through regulation of translation [112]. An interaction between pY478 ezrin and Fes kinase influencing actin cytoskeleton at cell–cell contacts and initiating cell scattering has been reported [113]. Fes contributes to neurite outgrowth in PC12 cells [114, 115], phosphorylates actin-regulatory proteins, but was also reported to interact with MT and regulate their dynamics [116, 117].

VEGFD administration, which leads to a reduction in the phosphorylation state of Y478 ezrin, interfered with the activity-induced increase of new dendrites; the effect was even more pronounced when the Y478F phosphorylation deficient mutant was expressed in neurons. A similar pattern was also observed for the increased elimination of activity-dependent newly formed dendrites. Notably, VEGFD prevented existing dendrites from elongating in response to sustained synaptic activity, but Y478-expressing neurons did not differ from control cultures in this regard. Nevertheless, the overexpression of WT and Y478F ezrin per se significantly increases dendrite arborization even in absence of stimuli to prompt remodeling. As such, certain aspects of dendrite dynamics, such as elongation and formation, may have already reached a plateau level and neurons fail to further increase them. Interestingly, overexpression of both WT and Y478F ezrin caused, under resting conditions, a partial down-regulation in both *VEGFD* and *VEGFR3* expression levels; an aspect suggestive of a feed forward mechanism supporting dendrite outgrowth.

In this study, we identified VEGFD signaling as a key regulator of the balance between dendritic maintenance and plasticity. As almost all disorders of the nervous system have been linked to various forms of structural aberrations, structural plasticity and stability of the established connections coexist within a fragile equilibrium. The identification and functional characterization of the regulatory mechanisms governing structure may therefore have far-reaching implications.

### Supplementary Information

Below is the link to the electronic supplementary material.

Supplementary file1 (XLSX 389 KB)

Supplementary file2 (PDF 8819 KB)

## Data Availability

All data are available in a source file and upon request. Inquiries regarding the data in this study should be addressed to the corresponding author.

## Abbreviations

ABP: Actin binding protein
AFM: Atomic force microscopy
Bic: Bicuculline
BDNF: Brain derived neurotrophic factor
CFC: Contextual and cued fear conditioning
DIV: Days in vitro
DARPP-32: Dopamine/adenosine-3′,5′-monophosphate-regulated phosphoprotein 32
ECL: Enhanced chemiluminescence
ESARE: Enhanced synaptic activity-regulated element
F-actin: Filamentous actin
FRAP: Fluorescence recovery after photobleaching
GAPDH: Glycerinaldehyd-3-phosphat-dehydrogenase
GFP: Green fluorescent protein
HBS: HEPES-buffered saline solution
hrGFP: Humanized *Renilla reniformis* GFP
hsyn: Human synapsin
mTOR: Mammalian target of rapamycin
MT: Microtubule
MAP: Microtubule associated proteins
EB3: Microtubule plus-end-binding protein 3
PKA: Protein kinase A
PP1: Protein phosphatase 1
PLA: Proximity ligation assay
QRT-PCR: Real-time quantitative polymerase chain reaction
rAAVs: Recombinant adeno-associated viruses
rVEGFA/-C/-D: Recombinant VEGFA/-C/-D
STEP: Striatal-enriched protein tyrosine phosphatase
VEGFA/-C/-D: Vascular endothelial growth factor A/C/D
gusb: β-Glucuronidase
