# Supplementary material for: VEGFD signaling balances stability and activity-dependent structural plasticity of dendrites
Source: Cell Mol Life Sci. 2024 Aug 19;81(1):354. doi: 10.1007/s00018-024-05357-2 (PMC11335284; doi:10.1007/s00018-024-05357-2)
Supplement: Supplementary file 2 — Supplementary file2 (PDF 8819 KB) [file 18_2024_5357_MOESM2_ESM.pdf]

SUPPLEMENTARY INFORMATION

VEGFD signaling balances stability and activity-dependent structural plasticity of dendrites

Bahar Aksan[1], Ann-Kristin Kenkel[1], Jing Yan[1], Javier Sánchez Romero[1], Dimitris Missirlis[2] and Daniela Mauceri[1, 3] *

Cellular and Molecular Life Sciences

[1] Department of Neurobiology, Interdisciplinary Centre for Neurosciences (IZN), Heidelberg University, INF 366, 69120 Heidelberg, Germany

[2] Max-Planck-Institute for Medical Research, Dept. of Cellular Biophysics, Jahnstraße 29, 69120 Heidelberg, Germany

[3] Institute of Anatomy and Cell Biology, Dept. Molecular and Cellular Neuroscience, University of Marburg, Robert-Koch-Str. 8, 35032 Marburg, Germany

* To whom correspondence should be addressed.

Prof. Dr. Daniela Mauceri

E-mail: mauceri@nbio.uni-heidelberg.de; mauceri@uni-marburg.de

This document contains:
Supplementary Figures 1-4 and relative legends
Supplementary methods

Supplementary fig. 1

Synaptic activity-dependent structural remodeling requires VEGFR3 activity and is unaffected by VEGFC.

a Representative images of ESARE>VEGFD-HA-hsyn>tDimer-transfected cultured hippocampal neurons with or without bicuculline (Bic) treatment for 24 h. HA-tag was detected immunocytochemically. Scale bar = 50 µm. **b** Representative immunoblots of cultured hippocampal neurons infected with rAAV-ESARE>VEGFD-HA or rAAV-

ESARE>LacZ-HA with or without Bic treatment for 24 h. **c** QRT-PCR analysis of *VEGFD* mRNA expression in cultured hippocampal neurons infected with rAAV-ESARE>VEGFD-HA or rAAV-ESARE>LacZ-HA with or without Bic treatment for 24 h. One-way ANOVA followed by Bonferroni's post hoc test. N = 5 independent culture preparations.

d Proximity ligation assay (PLA) with antibodies against VEGFR3 and phospho-tyrosine of cultured hippocampal neurons with or without rVEGFD treatment for 2 h. Left side shows representative images of PLA signal in neurons treated as indicated. Nuclei were labelled with Hoechst. Scale bar = 20 µm. Lower panels show higher magnification of dendrites labeled with MAP2. Scale bar = 5 µm. Right side shows PLA cluster number normalized on the number of Hoechst-labelled cells and on control. One sample t-test. N = 5 independent culture preparations.

e-g Morphometric analyses of cultured hippocampal neurons transfected with hrGFP, with or without rVEGFD, rVEGFD-Elab (rVEGFD-Alt) or Bic treatment, or in combination for 24 h. **e** Representative images of neurons treated as indicated. hrGFP was used to visualize neurons. Scale bar = 20 µm. **f, g** Total dendritic length (**f**) and total number of intersections (**g**) of neurons treated as indicated. One- and Two-way ANOVA followed by Bonferroni's post hoc test. N = 11-12 neurons from 3 independent culture preparations.

h Morphometric analyses (total dendritic length) of cultured hippocampal neurons transfected with hrGFP, with or without rVEGFD, DC101 or Bic treatment, or in combination for 24 h. One-way ANOVA followed by Bonferroni's post hoc test N = 11-12 neurons from 3 independent culture preparations.

i Morphometric analyses (total dendritic length) of cultured hippocampal neurons transfected with hrGFP, with or without rVEGFD, rVEGFA or Bic treatment, or in combination for 24 h. One-way ANOVA followed by Bonferroni's post hoc test N = 12 neurons from 3 independent culture preparations.

j Morphometric analyses (total dendritic length) of cultured hippocampal neurons transfected with hrGFP, with or without rVEGFD, SAR131675 or Bic treatment, or in combination for 24 h. One-way ANOVA followed by Bonferroni's post hoc test N = 11-12 neurons from 3 independent culture preparations.

k-n Morphometric analyses of cultured hippocampal neurons transfected with hrGFP and with or without rVEGFC or Bic treatment, or both for 24 h. **k** Representative images of neurons transfected and treated as indicated. hrGFP was used to visualize neurons. Scale bar = 20 µm. **l-n** Total dendritic length (**l**), Sholl analysis (minimal significant p value is displayed) (**m**) and total number of intersections (**n**) of neurons treated as indicated. One- and Two-way ANOVA followed by Bonferroni's post hoc test (**l, n**), or Dunnett's post hoc test (**m**). N = 14-17 neurons from 3 independent culture preparations.

o, p Time-lapse imaging of neurite length for 24 h of hrGFP-transfected cultured hippocampal neurons with or without rVEGFC or Bic treatment, or both. **o** Schema of the experimental setup. **p** Relative total neurite length over time normalized on the first

timepoint and untreated control. Two-way ANOVA followed by Dunnett's post hoc test. N = 3 independent cultures.

Graphs represent mean ± SEM. Dots represent single values. Asterisks (*) refer to statistical comparisons between conditions and hashtags (#) to comparisons to basal values per condition. ****$p < 0.0001$; ***$p < 0.001$; **$p < 0.01$; *$p < 0.05$; ####$p < 0.0001$; ###$p < 0.001$; ##$p < 0.01$; #$p < 0.05$; ns $p>0.05$

Supplementary fig. 2

Nuclear calcium transients induced by neuronal activity are not affected by VEGFD.

a Representative images of jRGECO1alpha-NLS fluorescence in cultured hippocampal neurons prior to and after bicuculline (Bic) or Bic and rVEGFD administration. **b** Representative traces showing the time course of evoked Bic-induced nuclear calcium transients in neurons treated as indicated. Scale bar = 100 µm. **c, d** Mean inter-peak interval (**c**) and mean peak amplitude (**d**) during the first ten minutes following treatment. Two-tailed unpaired Student's t-test. N = 6-7 coverslips from 4 independent culture preparations.

Graphs represent mean ± SEM. Dots represent single values

Supplementary fig. 3

Src is not involved in VEGFD-mediated regulation of Y478 ezrin phosphorylation in neurons.

a, b Immunoblot analysis in cultured hippocampal neurons treated with DMSO (vector) or 1 µM or 10 µM PP2 for 1 h or 24 h. **a** Representative immunoblots of ezrin, phospho Y478 ezrin, phospho Akt and tubulin. **b** Phospho Akt, ezrin, phospho Y478 ezrin and its phospho/total protein ratio normalized on tubulin and control. One sample t-test. N = 3-5 independent culture preparations.

c, d Proximity ligation assay (PLA) with antibodies against ezrin and phospho Y478 ezrin in cultured hippocampal neurons treated with DMSO (vector) or 10 µM PP2 for 1 h. **c** Representative images of PLA signal in neurons treated as indicated. Nuclei were visualized with Hoechst. Scale bar = 20 µm. **d** PLA cluster number normalized on the number of Hoechst-labelled cells and on control. One sample t-test. N = 5 independent culture preparations.

e Relative phosphorylation level of phospho Ser75, Tyr216, Tyr 418 and Tyr529 Src derived from the cytoskeleton-based phosphoantibody array from cultured hippocampal neurons with or without treatment of rVEGFD for 15 min or 2h (see also main figure 6). One-way ANOVA followed by Dunnett's post hoc test. N = 3 independent culture preparations.

f, g Immunoblot analyses of phospho Src protein expression in cultured hippocampal neurons with or without treatment of rVEGFD for 15 min. **f** Representative immunoblots of pSrc and tubulin. pSrc (**g**) levels normalized to tubulin expression and control. One sample t-test. N = 4 independent culture preparations.

h, i Immunoblot analyses of phospho Src protein expression in cultured hippocampal neurons with or without treatment of rVEGFD for 2 or 24 h. **h** Representative immunoblots of pSrc and tubulin. **i** pSrc levels normalized to tubulin expression and control. One-way ANOVA followed by Dunnett's post hoc test. N = 4 independent culture preparations.

j, k Immunoblot analyses of phospho NFATc1 protein expression in cultured hippocampal neurons with or without treatment of rVEGFD for 15 min. **j** Representative immunoblots of phospho NFATc1 and tubulin. **k** Phospho NFATc1 levels normalized to tubulin expression and control. One sample t-test. N = 3 independent culture preparations.

l Free phosphate released by a phosphopeptide substrate and indicative of calcineurin activity in homogenates from cultured hippocampal neurons with or without treatment of rVEGFD for 15 min. Two-tailed unpaired Student's t-test. N = 3 independent culture preparations.

Graphs represent mean ± SEM. Dots represent single values. ***p < 0.001; *p < 0.05

Supplementary fig. 4

Overexpression of ezrin affects VEGFD and VEGFR3 expression.

a-b QRT-PCR analysis of *VEGFD* (**a**) and *VEGFR3/Flt4* (**b**) mRNA expression levels in cultured hippocampal neurons infected with rAAV-LacZ, rAAV-ezrin WT or rAAV-ezrin Y478F. Values are normalized to LacZ-infected cultures. One-way ANOVA followed by Dunnett's post-hoc test. N = 5 independent culture preparations.

Graphs represent mean ± SEM. Dots represent single values. * p<0.05; **** p< <0.0001; ns p>0.05

Supplementary methods:

Calcium imaging

Nuclear calcium imaging was performed on rAAV-CaMKII>jRGECO1alpha-NLS-infected neurons [1] at DIV 10 in a HEPES-buffered saline solution (HBS) containing: 140 mM NaCl, 2.5 mM KCl, 1.0 mM $MgCl_2$, 2.0 mM $CaCl_2$, 10 mM HEPES, 1.0 mM glycine, 35.6 mM D-glucose, and 0.5 mM Na-pyruvate. Images were acquired at a widefield microscope (BX51W1, Olympus) with a 20x water-immersion objective with a frequency of 1 Hz. Fluorescence excitation was set at 570 ± 10 nm and provided by an LED light source (CoolLED pE-4000). jRGECO1alpha fluorescence was filtered at 620 ± 30 nm.

After 5 min of baseline recording, neurons were treated with 50 µM Bic or 100 ng/ml rVEGFD and 50 µM Bic and imaged for another 10 min.

Data were collected using proprietary software (VisiView, Visitron Systems), and analyzed using Fiji and IgorPro (WaveMetrics). Calcium signals in regions of interest drawn around individual nuclei were quantified as the baseline intensity-normalized change in fluorescence over time, $\Delta F/F = (F - F_{baseline})/F_{baseline}$. Calcium transients were quantified in terms of the mean peak amplitude and the mean inter-peak interval following the start of the treatment.

Calcineurin activity

Calcineurin activity was measured on lysates of cultured neurons using a specific Calcineurin Cellular Activity Assay Kit (Enzo Life Sciences) according to the manufacturer's protocol. In brief, after treatments, cultures were harvested in lysis buffer containing proteases inhibitors. Before proceeding with the assay, samples were checked for the presence of free phosphate which may affect the subsequent measurements. The method relies on the RII phosphopeptide substrate - known for its specificity for calcineurin- and measures the amount of released phosphate from the substrate via the Malachite green assay. Recombinant calcineurin was included as positive control. A calibration curve was created for each measurement, and absorbance values were used to calculate the amount of free phosphate from a standard curve line-fit in Prism software.

References cited in this document

1. Litke C, Hagenston AM, Kenkel A-K, Paldy E, Lu J, Kuner R, Mauceri D (2022) Organic anion transporter 1 is an HDAC4-regulated mediator of nociceptive hypersensitivity in mice. Nat Commun 13:875. https://doi.org/10.1038/s41467-022-28357-x

Suppl. fig. 1

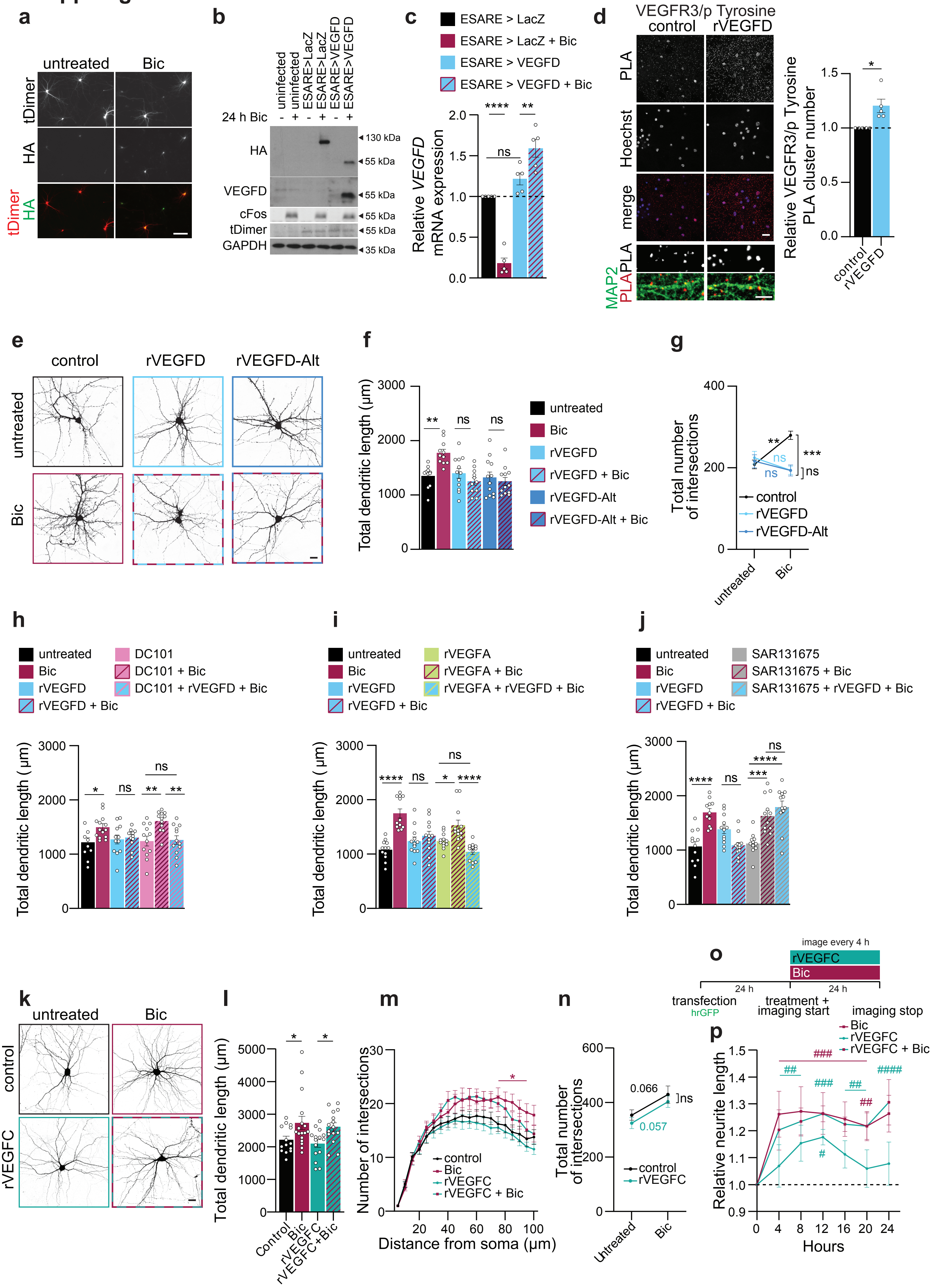

a
untreated
Bic
tDimer
HA
tDimer HA
b
uninfected
uninfected
ESARE>LacZ
ESARE>LacZ
ESARE>VEGFD
ESARE>VEGFD
24 h Bic
HA
VEGFD
cFos
tDimer
GAPDH
130 kDa
55 kDa
55 kDa
55 kDa
55 kDa
35 kDa
c
ESARE > LacZ
ESARE > LacZ + Bic
ESARE > VEGFD
ESARE > VEGFD + Bic
Relative VEGFD mRNA expression
d
VEGFR3/p Tyrosine
control
rVEGFD
PLA
Hoechst
merge
MAP2 PLA
Relative VEGFR3/p Tyrosine PLA cluster number
e
control
rVEGFD
rVEGFD-Alt
untreated
Bic
f
Total dendritic length (µm)
untreated
Bic
rVEGFD
rVEGFD + Bic
rVEGFD-Alt
rVEGFD-Alt + Bic
g
Total number of intersections
control
rVEGFD
rVEGFD-Alt
untreated
Bic
h
untreated
Bic
rVEGFD
rVEGFD + Bic
DC101
DC101 + Bic
DC101 + rVEGFD + Bic
i
rVEGFA
rVEGFA + Bic
rVEGFA + rVEGFD + Bic
j
SAR131675
SAR131675 + Bic
SAR131675 + rVEGFD + Bic
k
untreated
Bic
control
rVEGFC
l
Total dendritic length (µm)
Control
Bic
rVEGFC
rVEGFC+Bic
m
Number of intersections
Distance from soma (µm)
control
Bic
rVEGFC
rVEGFC + Bic
n
Total number of intersections
0.066
0.057
ns
control
rVEGFC
Untreated
Bic
o
image every 4 h
rVEGFC
Bic
24 h
24 h
transfection
hrGFP
treatment + imaging start
imaging stop
p
Bic
rVEGFC
rVEGFC + Bic
Relative neurite length
Hours

Suppl. fig. 2

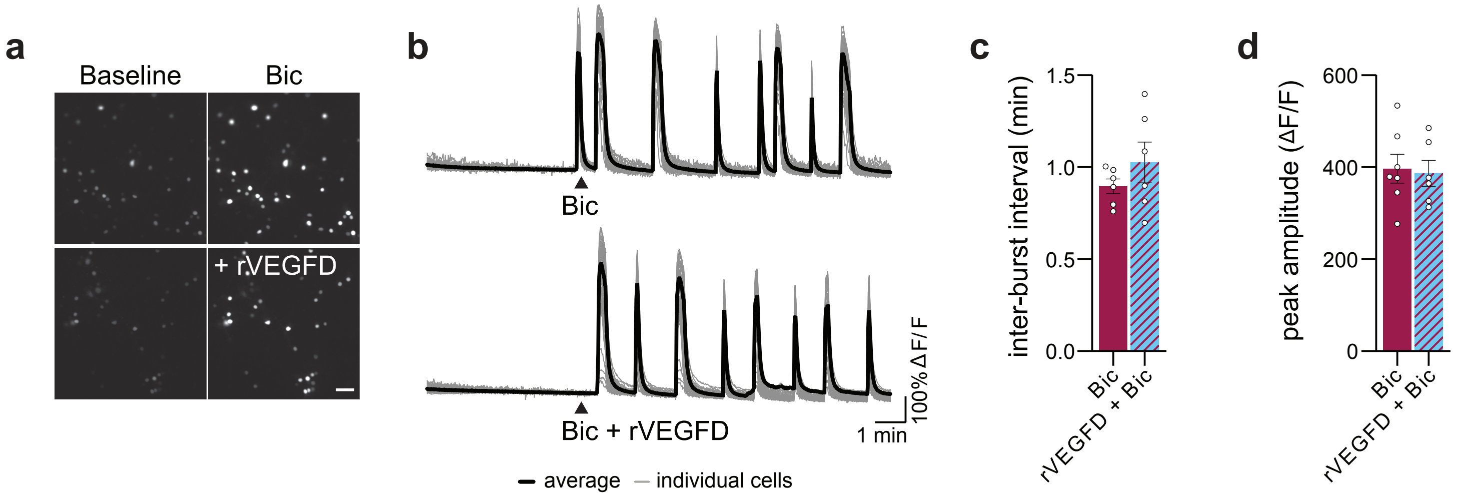
a
Baseline
Bic
+ rVEGFD
b
Bic
Bic + rVEGFD
100% ΔF/F
1 min
average
individual cells
c
inter-burst interval (min)
1.5
1.0
0.5
0.0
Bic
rVEGFD + Bic
d
peak amplitude (ΔF/F)
600
400
200
0
Bic
rVEGFD + Bic

Suppl. fig. 3

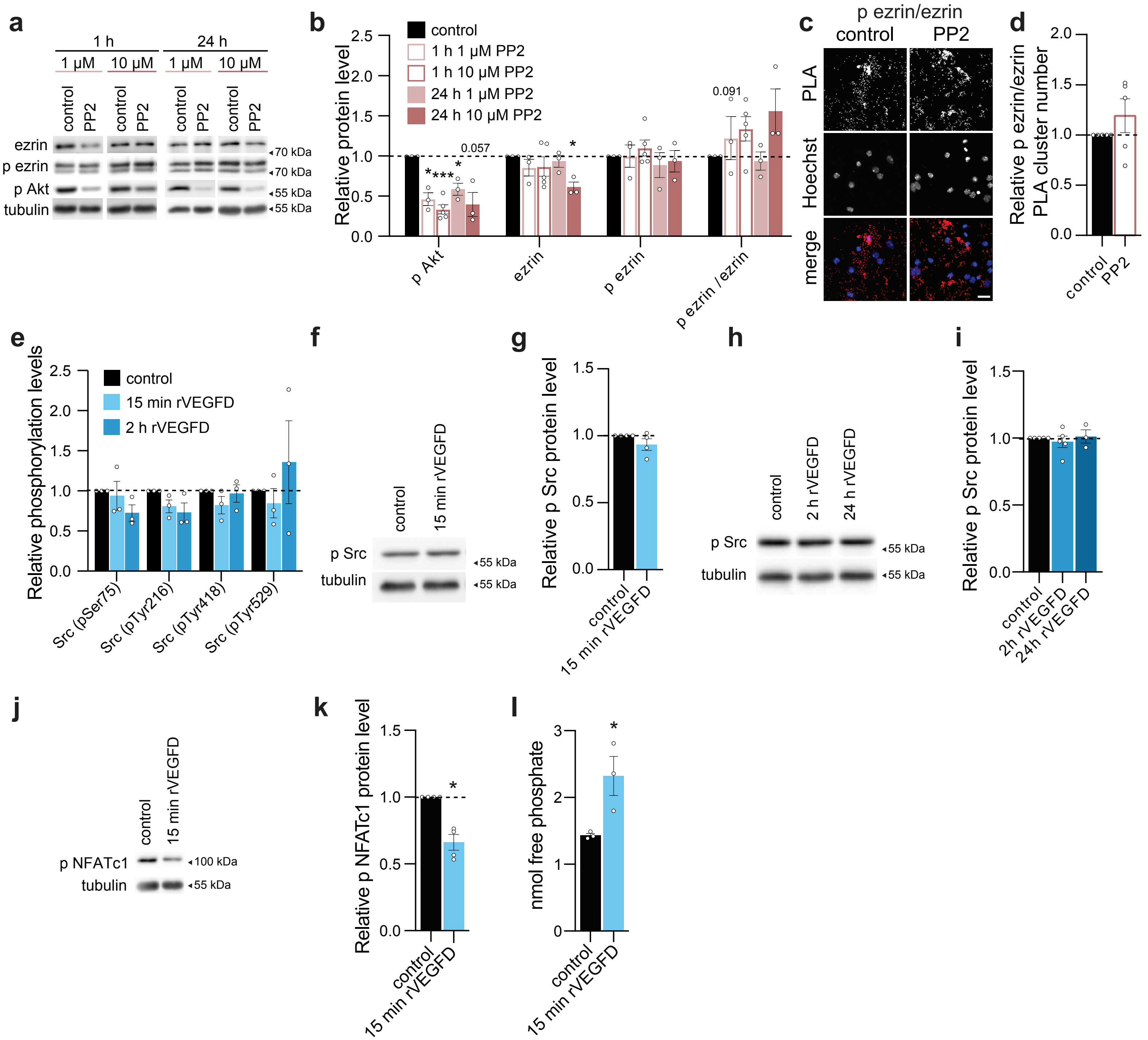
a
1 h
24 h
1 µM
10 µM
control
PP2
ezrin
p ezrin
p Akt
tubulin
70 kDa
55 kDa
b
control
1 h 1 µM PP2
1 h 10 µM PP2
24 h 1 µM PP2
24 h 10 µM PP2
Relative protein level
0.057
0.091
p Akt
ezrin
p ezrin
p ezrin /ezrin
c
p ezrin/ezrin
control
PP2
PLA
Hoechst
merge
d
Relative p ezrin/ezrin PLA cluster number
e
control
15 min rVEGFD
2 h rVEGFD
Relative phosphorylation levels
Src (pSer75)
Src (pTyr216)
Src (pTyr418)
Src (pTyr529)
f
15 min rVEGFD
p Src
55 kDa
g
Relative p Src protein level
h
2 h rVEGFD
24 h rVEGFD
i
2h rVEGFD
24h rVEGFD
j
p NFATc1
100 kDa
k
Relative p NFATc1 protein level
l
nmol free phosphate

Suppl. fig. 4

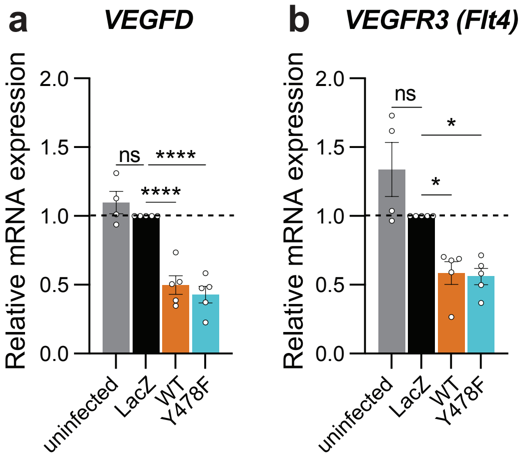

a
VEGFD
Relative mRNA expression
2.0
1.5
1.0
0.5
0.0
ns
****
****
uninfected
LacZ
WT
Y478F
b
VEGFR3 (Flt4)
Relative mRNA expression
2.0
1.5
1.0
0.5
0.0
ns
*
*
uninfected
LacZ
WT
Y478F
